# The chromosome‐scale genomes of *Dipterocarpus turbinatus* and *Hopea hainanensis* (Dipterocarpaceae) provide insights into fragrant oleoresin biosynthesis and hardwood formation

**DOI:** 10.1111/pbi.13735

**Published:** 2021-12-15

**Authors:** Sibo Wang, Hongping Liang, Hongli Wang, Linzhou Li, Yan Xu, Yang Liu, Min Liu, Jinpu Wei, Tao Ma, Cheng Le, Jinlong Yang, Chengzhong He, Jie Liu, Jianming Zhao, Yuxian Zhao, Michael Lisby, Sunil Kumar Sahu, Huan Liu

**Affiliations:** ^1^ State Key Laboratory of Agricultural Genomics BGI‐Shenzhen Shenzhen China; ^2^ College of Life Sciences University of Chinese Academy of Sciences Beijing China; ^3^ Department of Biotechnology and Biomedicine Technical University of Denmark Lyngby Denmark; ^4^ Key Laboratory of Bio‐resource and Eco‐Environment of Ministry of Education College of Life Sciences Sichuan University Chengdu China; ^5^ BGI‐Yunnan, BGI‐Shenzhen Yunnan China; ^6^ College of Forensic Science Xi'an Jiaotong University Xi'an China; ^7^ Southwest Forestry University Kunming, Yunnan China; ^8^ Forestry Bureau of Ruili Yunnan Dehong, Ruili China; ^9^ Chinese Academy of Forestry Beijing China; ^10^ Department of Biology University of Copenhagen Copenhagen Denmark

**Keywords:** genome, long reads, Dipterocarpaceae, whole‐genome duplication, Evolution, fragrance, oleoresin, wood formation

## Abstract

Dipterocarpaceae are typical tropical plants (dipterocarp forests) that are famous for their high economic value because of their production of fragrant oleoresins, top‐quality timber and usage in traditional Chinese medicine. Currently, the lack of Dipterocarpaceae genomes has been a limiting factor to decipher the fragrant oleoresin biosynthesis and gain evolutionary insights into high‐quality wood formation in Dipterocarpaceae. We generated chromosome‐level genome assemblies for two representative Dipterocarpaceae species *viz*. *Dipterocarpus turbinatus* Gaertn. f. and *Hopea hainanensis* Merr. et Chun. Our whole‐genome duplication (WGD) analysis revealed that Dipterocarpaceae underwent a shared WGD event, which showed significant impacts on increased copy numbers of genes related to the biosynthesis of terpene, *BAHD* acyltransferases, fatty acid and benzenoid/phenylpropanoid, which probably confer to the formation of their characteristic fragrant oleoresin. Additionally, compared with common soft wood plants, the expansion of gene families was also found to be associated with wood formation, such as in *CESA* (cellulose synthase), *CSLE* (cellulose synthase‐like protein E), laccase and peroxidase in Dipterocarpaceae genomes, which might also contribute to the formation of harder, stronger and high‐density timbers. Finally, an integrative analysis on a combination of genomic, transcriptomic and metabolic data from different tissues provided further insights into the molecular basis of fragrant oleoresins biosynthesis and high‐quality wood formation of Dipterocarpaceae. Our study contributes the first two representative genomes for Dipterocarpaceae, which are valuable genetic resources for further researches on the fragrant oleoresins and superior‐quality timber, genome‐assisted breeding and improvement, and conservation biology of this family.

## Introduction

Dipterocarpaceae plants play an important ecological role in studying the succession of forest communities because they are representative tropical tree species of pantropical rainforests (Sasaki, 2006). The Dipterocarpaceae family belongs to the order of the Malvales and consists of 16 genera and about 695 known species around the world (Appanah and Turnbull, 1998; Christenhusz and Byng, 2016), which are mainly distributed in tropical lowland wet rainforest area, from South America to Africa (Seychelles), and India, as well as Southeast Asia areas, *that is* China, Indonesia, Malaysia and Philippines (Appanah and Turnbull, 1998).

**Figure 1:**
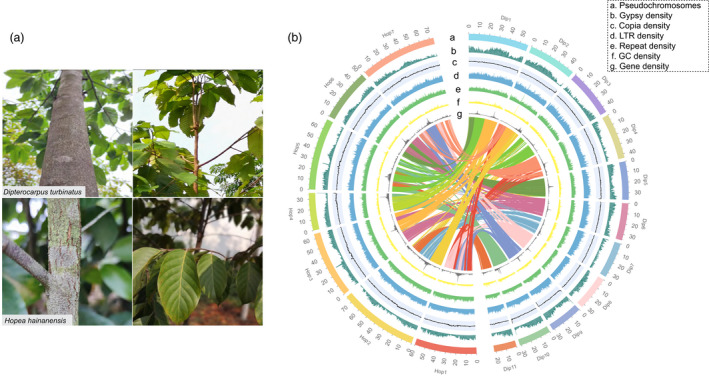
Morphology and genome features of *D. turbinatus* and *H. hainanensis*. (a) Leaf and stem of *D. turbinatus and H. hainanensis*, respectively. (b) The genomic landscape of *D. turbinatus and H. hainanensis*.

Dipterocarpaceae plants are of high economic value due to the presence of unique fragrant oleoresins, high‐quality timber and bioactive components for the preparation of traditional Chinese medicines (Dyrmose *et al*., 2017; Rana *et al*., 2010; Shen *et al*., 2017). The *Dipterocarpus* species serves as the principal source of fragrant oleoresins, which are present primarily in their tree trunk, and is a mixture of amorphous polymer compounds (Appanah and Turnbull, 1998). Other Dipterocarpaceae genera also produce fragrant oleoresins but are not that important, such as *Shorea*, *Vatica*, *Hopea*, *Dryobalanops* and *Parashorea* (Anakarfjärd and Kegl, 1998; Appanah and Turnbull, 1998). A famous oleoresin is procured from *D. turbinatus* wood (Figure 1a), which is the principal source to produce ‘keruing’ through the distillation and purification of fragrant oleoresins and also a famous perfumery over the world (Appanah and Turnbull, 1998; Aslam and Ahmad, 2015). The fragrance of oleoresins is good for health, as documented in ancient Chinese medical literature, such as inducing resuscitation, clearing away heat and detoxifying, relief of swelling, pain and tumescence (Peter and Babu, 2012; Zhou and Yang, 2017). The aromatic resins secreted from *D. turbinatus* are treated as ‘panacea’ for treatments of rheumatism, stubborn eczema and other skin diseases in Xishuangbanna of China (Zhou and Ren, 2007). Modern pharmacological and chemical studies also support the potential medicinal value of fragrant resins of Dipterocarpaceae since it is featured with antioxidant, antibacterial, anti‐fungi and anti‐inflammatory activities (Aslam *et al*., 2015; Yongram *et al*., 2019). The composition of oleoresins extracted from Dipterocarpaceae is exceedingly complex, including dozens of chemical compounds (Kamariyah *et al*., 2012); however, the main active compounds present in aromatic oleoresins of Dipterocarpaceae are various terpenoids (Kamariyah *et al*., 2012; Messer *et al*., 1990). Notably, it consists of distinct sesquiterpenes, gurjunene, which may be one of the main components that contribute to the unique fragrance of Dipterocarpaceae (Appanah and Turnbull, 1998).

Additionally, Dipterocarpaceae are highly demanded in the plywood industry in tropical Asia. The wood of Dipterocarpaceae is hard and tight with fine texture and strong moisture resistance (Rana *et al*., 2009). Especially, *Hopea hainanensis* (Figure 1a) is the wood, which grows much slower and needs hundreds of years to attain full maturity (Appanah and Turnbull, 1998; Oldfield and Lusty, 1998). However, its wood is extremely precious and famous for high strength, corrosion and insect resistance, durable and long‐lasting (Appanah and Turnbull, 1998). The wood of Dipterocarpaceae is widely used as bridge, ship and furniture construction materials (Appanah and Turnbull, 1998).

Many species of Dipterocarpaceae such as *Dipterocarpus* and *Hopea* are now endangered (Oldfield *et al*., 1998), mainly because of their low genetic diversity and overconsumption (Meng and Xu, 2005). There are a large number of inbreeds in the population of Dipterocarpaceae, resulting in a large loss of genetic variation of this species (Appanah and Turnbull, 1998; Meng and Xu, 2005; Zhou and Yang, 2017). Additionally, the overexploitation of natural populations and destruction of wild habitat further resulted in a dramatic decline of the population of Dipterocarpaceae in the past decades (Meng and Xu, 2005). Considering the increasing demands in the market of fragrant resins and timber of Dipterocarpaceae and decreasing population in many Dipterocarpaceae plants, it is important to interrogate the genomic background to explore the genome feature of Dipterocarpaceae and to accelerate genome‐assisted improvement in breeding systems.

Although several woody and economically important plant genomes have been reported earlier (Baek *et al*., 2018; Butkhup *et al*., 2011; Chang *et al*., 2018; Fan *et al*., 2020, 2021; Hofmeister *et al*., 2020; Sahu *et al*., 2019; Shang *et al*., 2020b; Zhang *et al*., 2019), the lack of Dipterocarpaceae genome has been a limiting factor to decipher the fragrant oleoresin biosynthesis and gain evolutionary insights into high‐quality wood formation in Dipterocarpaceae. In this study, high‐quality chromosome‐level genome assemblies of *D. turbinatus* and *H. hainanensis* (Figure 1b) were obtained by combining the Oxford Nanopore long‐read sequencing and Hi‐C scaffolding data. Comparative analyses of the *D. turbinatus* and *H. hainanensis* genomes with the other representative fragrant plants and timber trees provided an impetus to explore the evolution and differentiation mechanisms of fragrance biosynthesis and wood formation in Dipterocarpaceae. Through gene mining, exploration of transcriptome and metabolic data generated from different tissues, we present new insights into the molecular basis of fragrant resins biosynthesis and wood formation of Dipterocarpaceae. Our study shall serve as a foundation for future research on the evolution, phytochemistry and ecology of Dipterocarpaceae.

## Results

### Genome assembly and annotation

**Figure 2:**
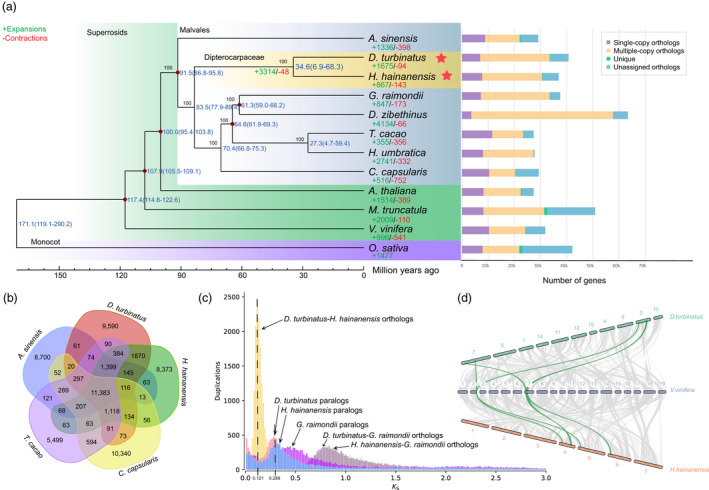
Evolution of the Dipterocarpaceae genomes and gene families. (a) Phylogenetic tree constructed by maximum likelihood based on the concatenation of single‐copy nuclear genes, the distribution of genes in each species is shown in the right panel. (b) Venn diagram of gene families in Dipterocarpaceae and other Malvales species. (c) The distributions frequencies of synonymous substitutions (*Ks*) for orthologs among two Dipterocarpaceae and *G. raimondii* (*D. turbinatus* (Dip), *H. hainanensis* (Hop) and *G. raimondii* (Goss). (d) Synteny patterns between genomic regions from two Dipterocarpaceae and *V. vinifera*. A collinear relationship is highlighted by one syntenic set shown in green colours.

The genomes sizes of *D. turbinatus* and *H. hainanensis* were estimated with K‐mer analysis to be 426.6 and 442.2 Mb, respectively (Additional file 1: Figure S1). The two genomes were sequenced and assembled using a combination of Oxford Nanopore long‐read, MGI‐SEQ short read and Hi‐C paired‐end read data. After primary assembly, correction, polishing and scaffolding, final assemblies of 421.2 and 434.3 Mb with contig N50 of 29 Mb and 9 Mb were obtained for *D. turbinatus* and *H. hainanensis*, respectively (Table 1). To refine both assemblies of *D. turbinatus* and *H. hainanensis*, their Hi‐C data were mapped to their draft assemblies; as a result, 99.9% and 99.6% of the entire genome sequences were effectively anchored, structured and oriented into 11 and 7 pseudochromosomes, respectively (Figure 1b, Figure 2, Additional file 1: Figure S2, Additional file 2: Table S1), which is in accordance with the reported number of chromosomes in somatic cells of *D. turbinatus* (2*n* = 22) and *H. hainanensis* (2*n* = 14) (Appanah and Turnbull, 1998). To assess the quality of the two genome assemblies, we performed BUSCO analysis and found that 90.7% and 91.4% complete eukaryotic conserved genes exist in the *D. turbinatus* and *H. hainanensis* genomes, respectively (Table 1 and Additional file 2: Table S2). Moreover, the LTR Assembly Index (LAI) value was 14.27 and 17.01 for *D. turbinatus* and *H. hainanensis*, respectively. Taken together, the above results indicated high degree of contiguity and completeness of the two Dipterocarpaceae genomes according to the current standards (Ellinghaus and Kurtz, 2008; Ou and Chen, 2018; Xu and Wang, 2007).

**Table 1:** Assembly and annotation features of the *D. turbinatus* and *H. hainanensis* genome

Species	*D. turbinatus*	*H. hainanensis*
Assembly feature		
Estimated genome size	426.64 Mb	442.20 Mb
Assembled genome size	421 171 415	434 306 036
GC content	32.71%	32.91%
N50 of contigs (bp)	245 261	6 614 031
N50 of scaffold (bp)	29 439 453	9 083 733
Total length of contig	421 013 775	434 305 978
Longest scaffold	49 593 048	30 910 234
Complete BUSCOs	90.70%	91.40%
Genome annotation		
Repeat region	46.40%	50.70%
Number of protein‐coding genes	40 707	36 967
Average length of transcripts (bp)	2459.20	2509.02
Average exon length (bp)	205.48	206.30
Average intron length (bp)	346.05	353.34
HIC		
Anchor size	420 837 890	432 493 699
Anchor rate	99.92%	99.58%
Number of pseudochromosomes	11	7
N50 of scaffold (bp)	37 048 376	69 706 040
Longest scaffold	58.2 M	76.9 M

Based on the high‐quality genomes of *D. turbinatus* and *H. hainanensis*, we found that *D. turbinatus* and *H. hainanensis* genomes contain 46.4% (195.5 Mb) and 52.4% (227.6 Mb) transposable elements, respectively (Additional file 2: Table S3). Long terminal repeats retrotransposons (LTR‐RTs) are the predominant components and comprise 26.5% and 31.7% of the genomes of *D. turbinatus* and *H. hainanensis*, respectively. Among the LTRs, the *Gypsy* elements were the most abundant in both genomes, followed by the *Copia* elements (Additional file 2: Table S3). The majority of intact LTRs of *Ty3/gypsy* and *Ty1/copia* in both Dipterocarpaceae genomes showed a similar pattern of insertion time of LTR‐RTs (Additional file 1: Figure S3), indicating LTR elements in Dipterocarpaceae underwent recent bursts and suggesting the major proportion of LTR‐RT elements in Dipterocarpaceae became active recently (Liu *et al*., 2021). By combining *ab initio*, homologue‐based and transcriptome‐based approaches, a total of 40 707 and 36 967 protein‐coding genes were predicted from *D. turbinatus* and *H. hainanensis* genomes, respectively, of which 88.6% and 89.7% shared homologs with annotated genes in public protein databases (Table 1 and Additional file 2: Table S4).

### Phylogenetics and genome evolution of Dipterocarpaceae

A phylogenetic tree was constructed for 12 selected plant species, including 8 representative species from Malvales, using genes extracted for 294 orthologous single‐copy nuclear genes. Molecular dating analysis suggests that Dipterocarpaceae diverged from the most recent common ancestor with Malvaceae at around 83.5 Mya, followed by the divergence of *Dipterocarpus* and *Hopea* at around 34.6 Mya (Figure 2a). The topology of our phylogenetic tree constructed by nuclear single‐copy gene contradicted with the previous study that used several discrete genes (Hernandez‐Gutierrez and Magallon, 2019). Their phylogeny showed a sister relationship between Thymelaeaceae and Dipterocarpaceae. However, their result also conflicts with other previous phylogenetic studies based on complete chloroplast genome sequences (Cvetković and Hinsinger, 2017; Lee *et al*., 2019; Yan *et al*., 2019). To further prove our nuclear phylogeny, we employed concatenation and coalescent methods by using RAxML and ASTRAL among 14 representative species (including 13 Malvales, transcriptome data from 1KP) (Leebens‐Mack *et al*., 2019), and both concatenation and coalescent phylogenetic trees showed consistent results with the phylogeny of Figure 2a with high bootstrap values (Additional file 1: Figure S4). To test whether there is cytonuclear discordance (the discordance between nuclear and organellar phylogenies) for Thymelaeaceae and Dipterocarpaceae, we assembled the chloroplast genomes of *D. turbinatus* and *H. hainanensis* in this study. Additionally, we also downloaded all the plastid genomes of Malvales from NCBI and performed phylogenetic analysis based on the complete chloroplast genome sequences by using RAxML and ASTRAL. Interestingly, both chloroplast genome‐based phylogenetic trees showed a similar topology that *D. turbinatus* and *H. hainanensis* of this study clustered with other Dipterocarpaceae species, and a sister relationship could be observed between Thymelaeaceae and Dipterocarpaceae (Additional file 1: Figure S4). Based on the phylogeny, we found 3,314 gene families expanded and 48 gene families contracted in the ancestor of Dipterocarpaceae. We also performed the KEGG enrichment for the expanded gene families in the common ancestor of the two newly sequenced species, which showed the expansion of mRNA surveillance pathway, Propanoate metabolism, Terpenoid backbone biosynthesis, and Pantothenate, CoA biosynthesis and Brassinosteroid biosynthesis pathways (Additional file 2: Table S5). From the gene families clustered with the other three representative species of the Malvales family (*Aquilaria sinensis*, *Theobroa cacao* and *Corchorus capsularis*), 19 833 gene families were Dipterocarpaceae‐specific while 11 383 gene families were shared among all the selected species of Malvales family (Figure 2c). Functional analysis showed that unique gene families in Dipterocarpaceae were preferentially enriched in the terms monoterpenoid biosynthesis, flavone and flavonol biosynthesis and fatty acid biosynthesis (Additional file 2: Table S6).

To investigate whether whole‐genome duplication (WGD) events happened in Dipterocarpaceae, the number of synonymous substitutions per synonymous site (Ks) was characterized of *D. turbinatus*, *H. hainanensis*, *C. capsularis*, *A. sinensis* and *G. raimondii*, respectively. *D. turbinatus* and *H. hainanensis* displayed almost the same *Ks* distributions of all paralogous gene pairs, and it unveiled evidence for a recent WGD event in the common ancestor of Dipterocarpaceae (Ks peak at ~0.29) after their divergence with Corchorus (Figure 2a and c). To provide additional evidence of shared WGD event between the *D. turbinatus* and *H. hainanensis*, we extracted paralogous pairs of *D. turbinatus* and *H. hainanensis* genes derived from their respective WGDs and constructed phylogenetic trees to further confirm that the WGD event was shared by *D. turbinatus* and *H. hainanensis* (Additional file 1: Figure S5). In addition, we detected a 2:2:1 syntenic relationship among *D. turbinatus*, *H. hainanensis* and *V. vinifera*, which provided additional evidence for a WGD event in the common ancestor of *Dipterocarpus* and *Hopea* (Figure 2d and Additional file 1: Figure S6). *That is*, a single *V. vinifera* region could be aligned to two genomic regions in the *D. turbinatus* or *H. hainanensis* genome (Figure 2e).

### Gene duplication contributes to the aromatic scent in Dipterocarpaceae

Gene duplication has long been regarded as one of the major driving forces in plant evolution, which may endow genes with potential sub‐functionalization and neo‐functionalization (Liu *et al*., 2021; Qiao *et al*., 2019). We identified a total of 33 230 and 29 176 duplicated genes from *D. turbinatus* and *H. hainanensis* genomes, respectively, which were classified into five categories, that is, the WGD duplicates, tandem duplicates (TD), transposed duplicates (TRD), proximal duplicates (PD) and dispersed duplicates (DD) (Additional file 2: Table S7). Both sequenced species in this study exhibited a similar trend concerning the numbers in each category of the duplicates.

**Figure 3:**
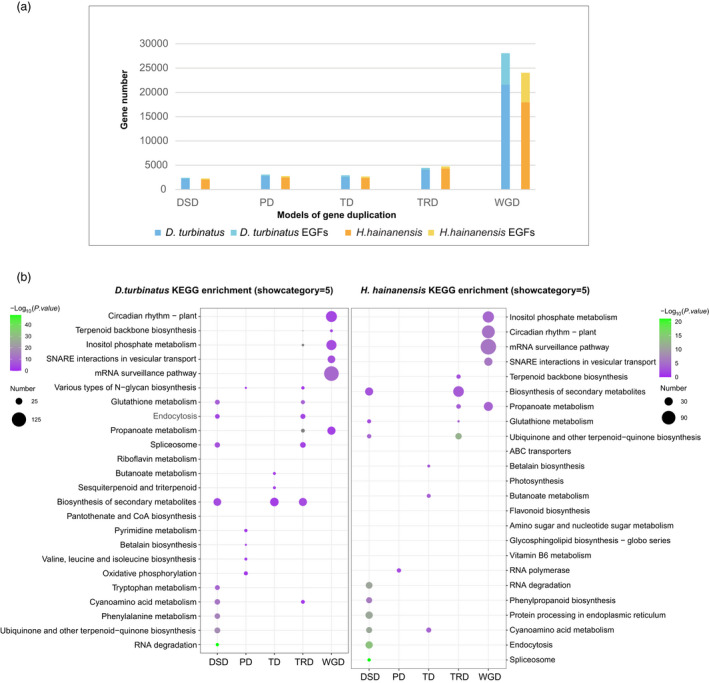
Gene duplication and evolution. (a) Stacked columns chart shows the number of gene duplication in various duplicated modes and gene duplication‐induced expanded gene number. (b) Functional enrichment of genes overlapping between expanded gene families and various modes of gene duplications.

Next, we analysed the duplication‐induced expansion in gene families by combining and overlapping each type of duplicated gene and expanded gene families (EGFs) (Figure 3a). WGD events contributed to the highest proportion of expansion of the gene families compared with other duplication types in both *D. turbinatus* and *H. hainanensis*. KEGG enrichment of expanded gene families in *D. turbinatus* and *H. hainanensis* indicated that, in *D. turbinatus*, the expansion of terpenoid backbone biosynthesis genes might be mainly caused by WGD events; the expansion of sesquiterpenoid and triterpenoid biosynthesis gene families was induced by TD; and the expansion of phenylpropanoid biosynthesis gene families derived from DSD events. In comparison, in *H. hainanensis*, TRD but WGD events contributed to the expansion of terpenoid backbone biosynthesis. Additionally, TD‐involved gene family in *H. hainanensis* was in response to the expansion of butanoate metabolism that is also related to fragrant volatiles (Butkhup *et al*., 2011). Similar to *D. turbinatus*, DSD‐involved gene family expansion also participated in the expansion of Phenylpropanoid biosynthesis genes in *H. hainanensis*. Taking these together, whole‐genome duplication and other types of duplication events (such as tandem duplications, dispersed duplicates) have significant impacts on the increase of copy numbers in genes related to terpene and phenylpropanoid biosynthesis that are involved in the generation of aromatic scent, which may contribute to the characteristic fragrance (aroma component) in Dipterocarpaceae (Additional file 2: Table S8).

### Evolution of terpene biosynthesis and regulation‐related genes

**Figure 4:**
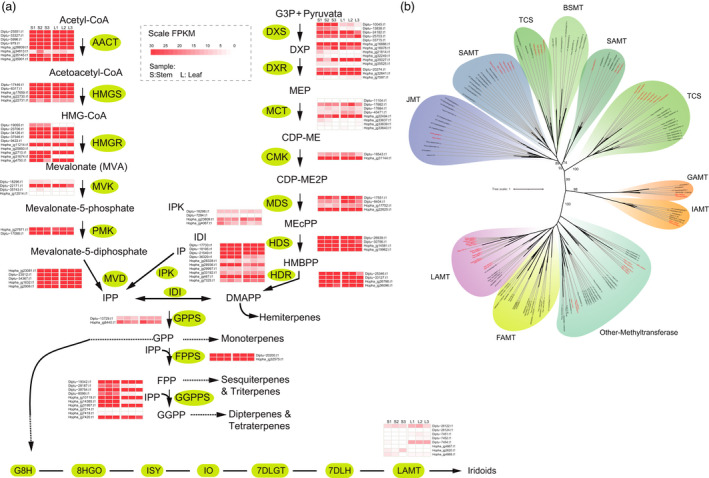
Biosynthetic pathway of MEP/MVA in Dipterocarpaceae. (a) MEP/MVA biosynthesis pathways in Dipterocarpaceae leaf and stem based on transcriptomic analyses. (b) Maximum likelihood phylogenetic tree showing the classification and copy number of SABATH family.

**Figure 5:**
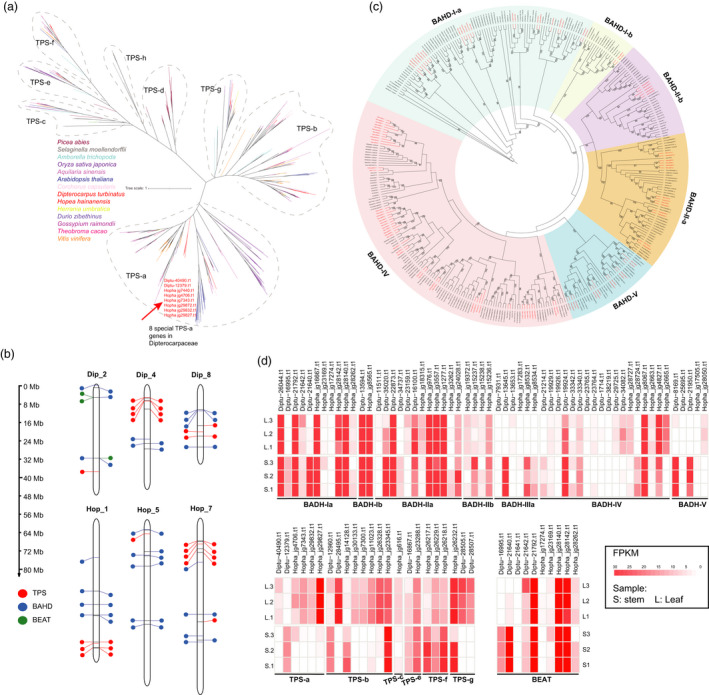
Phylogeny and expression level of TPS and BAHD families in Dipterocarpaceae. (a) Maximum likelihood phylogenetic tree showing the classification and number of TPS in Dipterocarpaceae. (b) Chromosomal distribution of TPS, BAHD and BEAT genes of two Dipterocarpaceae. (c) Maximum likelihood phylogenetic tree showing the classification and number of BAHD subfamilies in Dipterocarpaceae. (d) Comparison of the relative expression profiles of TPS and BAHD genes between leaf and stem tissues of two Dipterocarpaceae species.

The containing of volatile organic compounds is the main reason behind the process of unique fragrance of Dipterocarpaceae (Shang *et al*., 2020a; Yang *et al*., 2018). Previous metabolic studies on the oleoresins of *D. turbinatus* and *H. hainanensis* showed that dozens of distinct chemical constituents, including the azulon, α‐gurjunene, α‐copaene, δ‐elemene and borneol, exist in these two species (Zhou and Ren, 2007), which is consistent with our results of chemical detection analyses (Additional file 2: Table S8). Monoterpenes, sesquiterpenes and iridoids are usually generated via the 2‐C‐methyl‐D‐erythritol 4‐phosphate (MEP) pathway and the mevalonate (MVA) pathway (Figure 4a). A total of 55/58 associated genes from two pathways were found in the *D. turbinatus* and *H. hainanensis* genomes, respectively (Additional file 2: Table S9). Our results showed that the copy number of some of these genes was expanded in both *D. turbinatus* and *H. hainanensis* genomes. For example, genes of Isopentenyl‐diphosphate Delta‐isomerase, which is responsible for a reversible reaction between isopentenyl diphosphate and dimethylallyl diphosphate for sesquiterpene and monoterpene biosynthesis, expanded in both *D. turbinatus* and *H. hainanensis* (Additional file 2: Table S9). Notably, gurjunene (sesquiterpene) and borneol (monoterpene) might play key roles in the contribution of unique fragrance in Dipterocarpaceae (Appanah and Turnbull, 1998). The gene expression profile across wood and leaf tissues revealed transcripts of numerous MEP/MVA pathway genes, such as HMGR, DXS and GGPPS genes, were most abundant in wood tissue, which coincided with the fact that fragrant oleoresins only accumulated in tree wood but not leaves. (Figure 4a). Interestingly, we identified the number of *LAMT* homologs, which are key enzymes for biosynthesis of Iridoids, showed remarkable expansion in both *D. turbinatus* and *H. hainanensis* compared with other plants (Figure 4b). Terpene synthases (TPSs) are the vital enzymes responsible for the catalytic reaction in the MVA and MEP pathway to generate a basic skeleton of terpenoid compounds. We identified 20/21 TPS genes in *D. turbinatus* and *H. hainanensis*, respectively (Figure 5a and Additional file 1: Figure S7), which did not exhibit a remarkable difference in TPS number compared with other selected plants (Additional file 2: Table S10). Similar to previous results in other plants (Xu *et al*., 2020; Li, Wang, *et al*., 2021), many *TPS* genes in *D. turbinatus* and *H. hainanensis* also exhibited a tandem array, and these genes formed TPS gene clusters on chromosomes 4, 8, 10 and 1, 7 (Figure 5b and Additional file 1: Figure S8), suggesting these genes underwent recent tandem duplication events.

The *TPS‐a* and *TPS‐b* genes encode angiosperm‐specific sesquiterpene and monoterpene synthases predominantly (Li, Wang, *et al*., 2021; Shang *et al*., 2020a). Surprisingly, *D. turbinatus* only encodes two *TPS‐a* (Figure 5a), which is remarkably less than that of *H. hainanensis* and *Aquilaria sinensis*. Phylogenetic analysis of the TPS gene family from selected plants revealed that the *TPS‐a* from *D. turbinatus* and *H. hainanensis* forms an individual subclade (Figure 5a), suggesting lineage‐specific of *TPS‐a* genes might own unique function contribute to the sesquiterpenes (especially for the distinct sesquiterpenes of Dipterocarpaceae essential oil, *i.e*., alpha and beta gurjunene) accumulation of fragrant resins in *D. turbinatus* and *H. hainanensis*. The comparison of the expression level of TPS genes of *D. turbinatus* and *H. hainanensis* between wood and leaf tissues revealed that the expression levels of Diptu_12379.t1 (*TPS‐a*) and Diptu_12960.t1 (*TPS‐b*) were significantly higher in wood than leaves (Figure 5d), suggesting they might play a primary role in the biosynthesis of sesquiterpene and monoterpene, the main components of fragrant oleoresin of *D. turbinatus*. Terpenes are usually modified by BAHD acyltransferases to produce esters, which are involved in the synthesis of various flavours and fragrances in plants (Xu *et al*., 2020; Shang *et al*., 2020a). A total of 51 and 40 BAHD acyltransferases were identified in the genomes of *D. turbinatus* and *H. hainanensis*, respectively (Figure 5c and Additional file 2: Table S11). Phylogenetic analysis revealed the number of BAHD acyltransferases V type in both *D. turbinatus* and *H. hainanensis* was prominently higher than that of *A. sinensis*, *C. capsularis*, *V. vinifera* and *T. cacao*. By comparing the expression level between wood and leaf tissues in *D. turbinatus* and *H. hainanensis*, we found that many BAHD acyltransferase genes from BADH‐Ia, BADH‐IIIa and BADH‐V groups were highly expressed in wood than in leaf tissue of both *D. turbinatus* and *H. hainanensis*, indicating BAHD acyltransferases might also contribute to the unique aroma of Dipterocarpaceae (Figure 5d).

### Evolution of benzenoid/phenylpropanoid biosynthesis‐related genes

**Figure 6:**
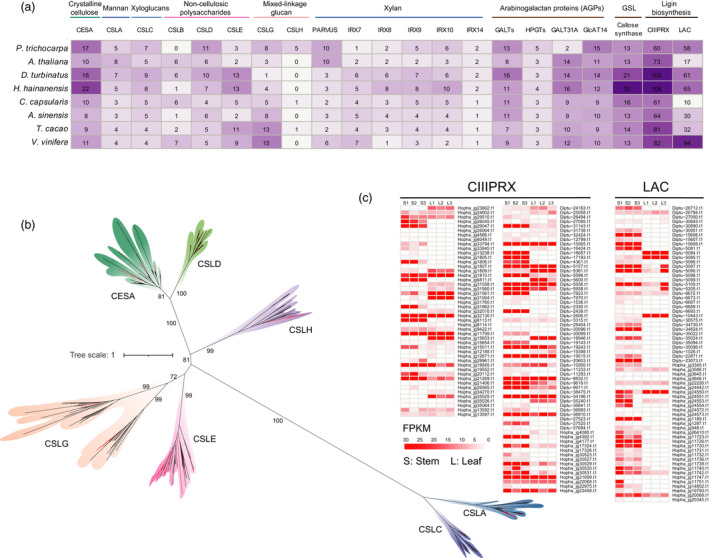
Evolution and expression of key genes involved in wood formation in Dipterocarpaceae. (a) The heat map shows a comparison of the numbers of key genes of related to cell wall formation and lignin metabolism among Dipterocarpaceae and representative plants. (b) Phylogenetic tree of the cellulose synthase (including cellulose synthase‐like) genes. (c) Comparison of the relative expression profiles of the Laccase and peroxidase between leaf and stem tissues of two Dipterocarpaceae species.

In plants, Phenylpropanoids and benzenoids are one of the largest class of compounds responsible for the fragrances (Li, Chen, *et al*., 2021; Zhao *et al*., 2017). We therefore investigated the expression of key genes involved in benzenoid/phenylpropanoid biosynthesis in both Dipterocarpaceae genomes (Additional file 2: Table S12). As phenylpropanoid biosynthesis and phenylalanine metabolism are two different metabolic pathways, and they share phenylalanine precursors (at the upstream), we considered both the pathway‐related genes to avoid the bias while performing the functional annotation, and found that while the majority of genes involved in phenylpropanoid biosynthesis exhibited higher expression, there were also many genes from phenylalanine synthesis that also displayed higher expression (Additional file 2: Table S13). We found that most of these genes showed remarkably higher level of expression in wood tissue than in leaf tissue in both *D. turbinatus* and *H. hainanensis* (Additional file 2: Table S13). acetyl‐CoA:benzyl alcohol acetyltransferase (*BEAT*) plays a key role in yielding benzyl acetate in plants for floral scent, and we identified 9/6 *BEAT* homologous genes in *D. turbinatus* and *H. hainanensis* (Additional file 2: Table S12). We found two BEATs displayed remarkably higher expression levels in stem tissue of *D. turbinatus* than in leaf tissue (Figure 5d), indicating a higher activity of benzyl acetate biosynthesis in the stem tissue for the aroma of *D. turbinatus*. The production of phenylpropanoid/benzenoid compounds in plants is related to the SABATH families (Xu *et al*., 2020). Phylogenetic analysis showed 29/24 SABATH homologs, including 1/1 *IAMT*, 1/1 *JMT*, 6/5 *SAMT*, 1/1 *GAMT*, 8/8 *LAMT*, 8/5 *TCS* and 6/3 others, existed in genomes of *D. turbinatus* and *H. hainanensis*, respectively (Figure 4, 6, 6 and Additional file 2: Table S14). Additionally, both the *COMT* and *ICMT*, belonging to the SAM‐binding methyltransferase superfamily, are thought to be involved in aromatic compound metabolism. Phylogenetic analysis showed both *D. turbinatus* and *H. hainanensis* own similar copy number of *COMT* and *ICMT* compared with other selected plants (Additional file 1: Figure S9 and Additional file 2: Table S14). However, the expression level of these genes in stem tissue from both *D. turbinatus* and *H. hainanensis* was mostly higher than their respective leaf tissue. Thus, transcriptomic data provided evidence for the higher activation of *COMT* and *ICMT* genes in stem tissue of two the Dipterocarpaceae species than in their leaf tissues (Additional file 2: Table S13).

### Hardwood formation in Dipterocarpaceae

Hardness, high strength and strong moisture resistance are the typical features of wood of Dipterocarpaceae plants (Appanah and Turnbull, 1998). To explore the genetic basis of the wood formation of Dipterocarpaceae, comparative genomics analyses on the genes related to the cell wall and lignin metabolic pathways were performed among the genomes of Dipterocarpaceae and other representative tree species characterized by relatively loose and soft wood texture (i.e. *Populus trichocarpa* and *A. sinensis*). *D. turbinatus* and *H. hainanensis* could encode 64/60 cellulose synthase (CESA) (including cellulose synthase‐like (CSL)), respectively, which is remarkably higher than the number of two representative softwood trees (*Populus trichocarpa* and *A. sinensis*) and other phylogenetic affiliation species (Figure 6a). Phylogenetic analysis indicated that the copy number of CESA and CSLE exhibited expansion in both *D. turbinatus* and *H. hainanensis* compared with that of *P. trichocarpa* and *A. sinensis* (Figure 6b). CESA is involved in the primary cell wall formation and considered as the most important enzyme involved in the synthesis of cellulose microfibrils in plant cells (Kumar and Turner, 2015). Interestingly, the number of laccase and peroxidase participating in the lignin metabolism, in both *D. turbinatus* and *H. hainanensis*, showed remarkable expansions compared with *P. trichocarpa* and *A. sinensis* featured with the softwood (Figure 6a). Additionally, the expression level of genes related to the lignin metabolism was compared between stem and leaf tissue in *D. turbinatus* and *H. hainanensis*, respectively. Most of the genes associated with the lignin metabolism showed higher expression in stem tissue of both *D. turbinatus* and *H. hainanensis* than their respective leaf tissue, reflecting these expanded lignin‐related genes might contribute to high lignin accumulation in stem tissues of both Dipterocarpaceae plants (Figure 6c). Thus, we anticipated that the expanded copy number of the CESA, CLSE, as well as the laccase and peroxidase in Dipterocarpaceae genomes might contribute to the evolutionary changes of the wood constitution (*i.e*. the ratio of cellulose microfibrils, hemicellulose and lignin molecule) and further result in the formation of harder, stronger and highly dense wood compared with those softwood plants.

### Transcription factors and phytohormone contribute to the regulation of fragrant resins biosynthesis and wood formation in Dipterocarpaceae plants

**Figure 7:**
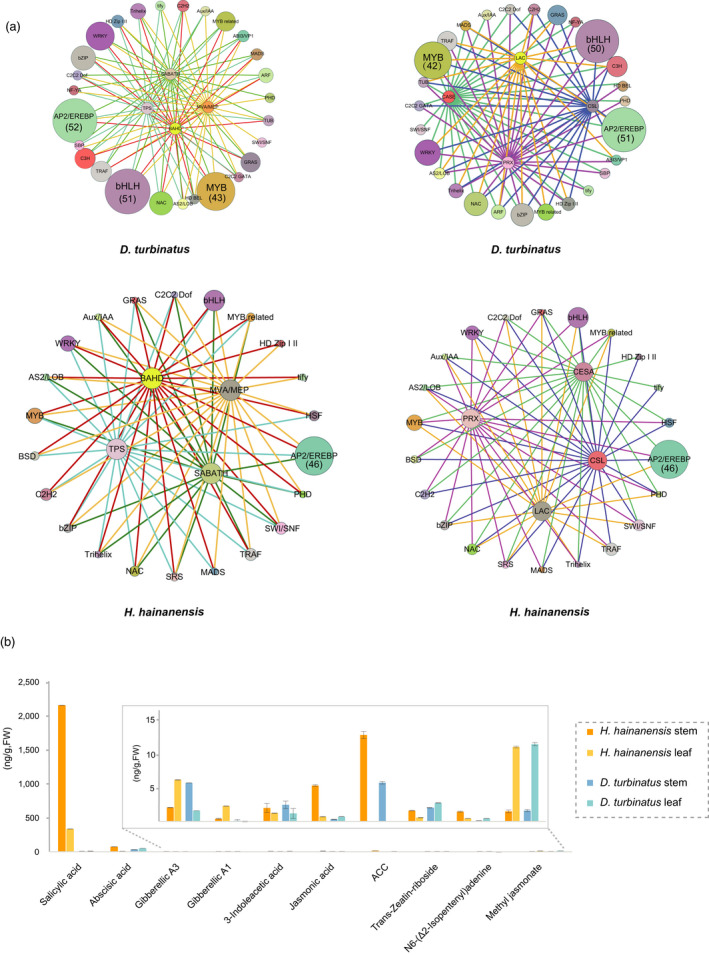
The regulation of fragrance and timber formation in Dipterocarpaceae plants. (a) Quantitation of eight plant phytohormones amounts in leaf and stem of two Dipterocarpaceae trees. (b) Co‐expression networks of fragrance formation‐related genes and transcription factors in two Dipterocarpaceae species (left panel). Co‐expression networks of cell wall metabolism and lignin‐related genes and transcription factors in two Dipterocarpaceae species (right panel), the gene number of highlighted TF were shown in the in brackets.

The formation of fragrant resins and hardwood does not rely on a variety of biochemical properties of enzymes. Many transcription factors (TFs) and phytohormones also participate in these complicated regulatory networks (Plomion and Leprovost, 2001; Shang *et al*., 2020a). A total of 2,401 and 2,397 TFs were respectively identified in *D. turbinatus* and *H. hainanensis*, which was remarkably higher than plants with relatively close relationships (Additional file 2: Table S15). To study the transcriptional regulatory networks of terpenoids biosynthesis and hardwood formation of Dipterocarpaceae wood, we performed a weighted correlation network analysis of transcript expression between TPSs and relative TFs as well as wood biosynthetic genes and relative TFs. In *D. turbinatus* stems, several *AP2/EREBP*, *bHLH* and *MYB* genes were shown to have a wide association in regulating fragrance‐related and wood growth‐related genes (Figure 7b). However, in the stem of *H. hainanensis*, the high number of *AP2/EREBP* exhibited a strong co‐relationship with both fragrance and wood growth‐related genes, which was comparatively fewer by *MYB* and *bHLH* with respect to *D. turbinatus*.

Comparing the various phytohormones content between stem and leaf, we found auxin, ethylene and N6‐(Δ2‐Isopentenyl) adenine (a naturally occurring cytokinin) in *D. turbinatus* and *H. hainanensis* both showed significantly higher accumulation in stem tissue than its leaf tissue (Figure 7a and Additional file 2: Table S16). However, other types of phytohormones displayed different patterns between *D. turbinatus* and *H. hainanensis*. Salicylic acid (SA), jasmonic acid (JA) and abscisic acid (ABA) exhibited higher accumulation in the stem tissue of *H. hainanensis* than its leaf tissue, while almost no difference between leaf and stem tissues for *D. turbinatus*. Gibberellin accumulation in *D. turbinatus* stem tissue was over twofold higher than its leaf tissue; conversely, Gibberellin was preferred to be produced in *H. hainanensis* leaf rather than its stem (Figure 7a). Thus, these phytohormones might contribute to the regulation of the physiologies of stem tissue between *D. turbinatus* and *H. hainanensis* in different ways.

## Discussion

Dipterocarpaceae are widely known trees (dipterocarp forests) in the tropics and predominate the international tropical timber market, and therefore play an important role in the economy of many Southeast Asian countries (Appanah and Turnbull, 1998). Besides a high value for its hardwood timber, the non‐timber species of Dipterocarpaceae are also wildly used in the perfume industry and pharmaceutical applications (Appanah and Turnbull, 1998). In this study, we present the nuclear genome assemblies of *D. turbinatus* and *H. hainanensis* at chromosome level by combining the long‐read sequences from Nanopore and Hi‐C data for super‐scaffolding. The first two Dipterocarpaceae genomes presented here provided a valuable opportunity to determine genome evolutionary signatures and elucidate the genetic basis of these high‐value metabolism products.

Phylogenomic analysis of a concatenation of 294 single‐copy nuclear genes from 12 representative species (including 8 Malvales) and 226 single‐copy nuclear genes from 14 representative species (including 13 Malvales) indicated that the two Dipterocarpaceae species *D. turbinatus* and *H. hainanensis* form a clade and are sister to Malvaceae, and a result is also supported by the coalescent analysis of nuclear genes. This relationship is consistent with previous studies from chloroplast genomes (Lee *et al*., 2019), but is in conflict with a previous result (Hernandez‐Gutierrez and Magallon, 2019), which recovered a sister relationship of Dipterocarpaceae and Thymelaeaceae on the basis of several discrete genes. Based on whole‐genome sequences of the two representative plants of Dipterocarpaceae, our analyses revealed that the common ancestor of Dipterocarpaceae experienced a shared WGD event after the divergence from jute species. Polyploidization event is one of important evolutionary formations that have the ability to contribute to species divergence and adaptation (Madlung, 2013; Scarpino and Levin, 2014); therefore, the occurrence of WGD event in the common ancestor of Dipterocarpaceae might have driven the species richness in Dipterocarpaceae. Dipterocarpus genus serves as the principal source to produce fragrant oleoresins from its wood, but other Dipterocarpaceae genera are with lesser production of fragrant oleoresins thus are less economically important (Appanah and Turnbull, 1998). The most famous oleoresin is procured from *D. turbinatus* wood, which is the principal source to produce perfumery over the world (Appanah and Turnbull, 1998). In consistent with previous studies (Aslam *et al*., 2015; Wang *et al*., 1992; Zhou and Ren, 2007), we also found several types of terpenoids in the wood of both *D. turbinatus* and *H. hainanensis* (Additional file 2: Table S8), which were the main fragrances contributor in Dipterocarpaceae. Gurjunene is a unique sesquiterpenes of Dipterocarpaceae which is responsible for the distinct fragrances of Dipterocarpaceae essential oils (Appanah and Turnbull, 1998). Interestingly, we found a few Dipterocarpaceae*‐*specific *TPS‐a* genes participating in sesquiterpene accumulation of fragrant resins in Dipterocarpaceae, especially for the *D. turbinatus*. However, only two Dipterocarpaceae*‐*specific *TPS‐a* genes were detected in its genome, indicating a diverse catalytic ability of sesquiterpene of *TPS‐a* in *D. turbinatus*. The production of terpenes is mainly regulated by the transcription level of TPS genes as well as its upstream pathway (MVA/MEP) genes (Li, Wang, *et al*., 2021). The results of the gene expression analyses revealed a dynamic expression of the TPS genes and MVA/MEP pathway‐related genes, which may be another explanation for the terpene diversification in the two Dipterocarpaceae plants. In addition to the monoterpenes, sesquiterpenes, the remarkable expansion of LAMTs in both *D. turbinatus* and *H. hainanensis* suggesting iridoids might play an essential role in the formation of Dipterocarpaceae's peculiar scent (Consortium, 2018). Additionally, other fragrance contributors, such as benzenoid/phenylpropanoid biosynthesis‐related genes, BAHD acyltransferases and SAM‐binding methyltransferase (*COMT* and *ICMT*), most displayed higher activity in stem tissue of *D. turbinatus* and *H. hainanensis* than their respective leaf tissues, further suggesting complicated fragrance composition in Dipterocarpaceae wood. Using genomic data, we classified various types of duplication‐induced gene family expansion. Functional enrichment of each type of duplication‐induced gene family expansion revealed that whole‐genome duplication and other types of duplication events have significant impacts on copy numbers of genes related to terpene, phenylpropanoid biosynthesis and lipid biosynthetic process (Additional file 2: Table S17) that are involved in aromatic oleoresin, which may contribute to the characteristic fragrance oleoresin in Dipterocarpaceae.

Wood is a mixture of polymers, partially composed of crystalline cellulose microfibrils and large amorphous hemicellulose and lignin molecules (Dhugga, 2012; Kumar and Turner, 2015). In wood, cellulose is one of the strongest polymers and hence is mainly responsible for the strength of the wood fibre (Rowell, 2012). Hemicelluloses are amorphous and thus easily hydrolysed into monomer sugars (Mota *et al*., 2018). However, hemicelluloses are embedded and interact with cellulose and lignin, which significantly increase the strength and toughness of the plant cell wall (Berglund *et al*., 2020). Generally, the packing density of the cell wall hemicelluloses is in a relatively greater proportion in hardwoods than in softwoods (Khatib, 2016). For tree plants, lignin could be distinguished into softwood lignin and hardwood lignin with different chemical compositions (Huang *et al*., 2012). Compared with representative softwood tree (*P. trichocarpa* and *A. sinensis*), Dipterocarpaceae own expanded CESA, CSLE, Laccase and peroxidase. Moreover, most of these genes exhibited higher expression in wood tissue than their expression status in leaves. CESA is involved in the primary cell wall formation and is thought to be the most important enzyme involved in the synthesis of cellulose microfibrils in plant cells (Kumar and Turner, 2015). Additionally, CSLE is proposed to be a Golgi‐localized beta‐glycan synthase that polymerizes the backbones of non‐cellulosic polysaccharides (hemicelluloses) of the plant cell wall (Dhugga, 2012). Increased copy numbers of CESA and CSLE in both Dipterocarpaceae genomes may boost the efficacy of catalytic reactions via dosage effects, resulting in increased metabolic activity towards cellulose microfibrils and hemicelluloses. Laccase is necessary and non‐redundant with peroxidase for lignin polymerization during vascular development in *Arabidopsis*. They are responsible for the catalysation of monolignols from corresponding p‐hydroxyphenyl (H), guaiacyl (G) and syringyl (S) lignin unit (Dixon and Barros, 2019; Wang *et al*., 2015). The expansion of cell wall‐related genes in Dipterocarpaceae genomes might contribute to the evolutionary changes of wood constitution, further inducing the formation of harder, stronger and denser wood.

Additionally, unique physiologies of Dipterocarpaceae wood may also necessitate complicated regulation by transcription factors and phytohormones. Ethylene, 3‐Indoleacetic acid and N6‐(Δ2‐Isopentenyl) adenine showed higher accumulation in wood of both *D. turbinatus* and *H. hainanensis* compared with their leaves. All three phytohormones were reported to affect the emission of terpenoids and wood formation/growth in other plants (Boncan *et al*., 2020; Savidge, 2003). Not all phytohormones showed a similar situation in both *D. turbinatus* and *H. hainanensis*. SA and JA displayed >sevenfold higher accumulation in *H. hainanensis* woods than its leaves, but they are not differentiated between woods and leaves of *D. turbinatus*. JA and SA both play important roles in transducing the activation of plant defence systems against pathogen attacks and responses to various abiotic stresses (McDowell and Dangl, 2000); therefore, the higher accumulation of SA and JA in the wood tissue of *H. hainanensis* might suggest its inherent defence ability against the pathogens. Notably, the remarkably expanded gene copy number of ‘Protein ENHANCED PSEUDOMONAS SUSCEPTIBILITY (EPS)’ in *H. hainanensis* genome compared with *D. turbinatus* (Additional file 2: Table S18) might also provide a clue to explain the higher SA level in *H. hainanensis*. Moreover, EPS encodes BAHD acyl transferase‐like protein has been shown to trigger SA accumulation and SA‐mediated resistance to virulent and avirulent pathogens in *A. thaliana* (Zheng *et al*., 2009). Unexpectedly, we found that both Dipterocarpaceae genomes underwent massive loss of resistance gene families related to immunity, such as leucine‐rich repeat receptor‐like protein kinases (LRR‐RLKs) and nucleotide‐binding leucine‐rich repeat (NBS‐LRR) gene families which were markedly lower than that in all the other species investigated (Additional file 2: Table S19). These results might suggest Dipterocarpaceae mainly rely on its complex oleoresin terpene defences against herbivores and pathogens mediated by phytohormones such as JA and SA to compensate for the huge loss of immunity‐related genes in their genomes (Celedon and Bohlmann, 2019; Nong *et al*., 2020). Moreover, the key components of Dipterocarpaceae oleoresin, such as gurjunene and borneol, have a slightly spicy smell, exhibiting higher activity in deterring aggressive insects (Li, Wang, *et al*., 2021).

*D. turbinatus* woods displayed ~fourfold higher GA3 accumulation than its leaves. GA3‐treated Cannabis exhibited an augmented HMGR activity of MVA pathway that is primarily essential for the synthesis of sesquiterpene (Mansouri and Asrar, 2009). GA3 could potentially have opposite effects on MEPs and MVA pathways, with stimulatory and inhibitory impacts on the key terpenoids produced via MVA and MEP pathways (Mansouri *et al*., 2009). Consequently, we hypothesized that the high concentration of GA3 found in *D. turbinatus* woods would activate its MVA activity and inhibit the MEP pathway, resulting in increased sesquiterpene production but not increased monoterpene production. Overall, based on our findings, we hypothesize that gene expression dynamics, duplication‐induced gene family expansion on aroma metabolism and cell wall associated genes may all contribute to the abundant feature in Dipterocarpaceae woods.

In summary, we presented high‐quality assemblies of *D. turbinatus* and *H. hainanensis*, the first two reference genomes from the Dipterocarpaceae family. The integration of multi‐omics data advanced our understanding of fragrant oleoresin biosynthesis and hardwood formation in Dipterocarpaceae plants. The two available complete Dipterocarpaceae genomes provided a fundamental resource for comparative genomic studies on the evolutionary mechanisms of secretion traits (fragrant oleoresin) and wood formation in these timber species at the genomic level, which will also be a valuable genetic resource for further research on the genome‐assisted breeding and improvement, and conservation biology of Dipterocarpaceae.

## Methods

### Genome and transcriptome sequencing

All plant materials of *Dipterocarpus turbinatus* Gaertn. f. (HCNGB_00001637) and *Hopea hainanensis* Merr. et Chun. (HCNGB_00001636) used in this study were collected from Ruili Botanical Garden (Yunnan, China). High‐quality DNA was extracted from fresh leaves by using QIAGEN^®^ Genomic kits, and the DNA quantification was checked by Nanodrop and Qubit. PromethION Nanopore sequencer with the long‐read DNA sequencing type was used for genome sequencing. The SQK_LSK109 Ligation Sequencing Kit was used to prepare the sequencing libraries. Finally, a total of 27 and 26 Gb pass reads were generated for *D. turbinatus* and *H. hainanensis*, respectively.

For short read Illumina sequencing, the genomic DNA was isolated from fresh leaves using a modified CTAB protocol (Sahu and Thangaraj, 2012). The extracted DNA was used to create four paired‐end libraries (170, 350, 500, and 800 bp) and four mate‐pair libraries (2, 6, 10 and 20 Kb) using the Illumina standard methods (San Diego, CA). Following that, the sequencing was performed by employing a whole‐genome shotgun sequencing approach on an Illumina HiSeq 2000 platform (San Diego).

### Estimation of the genome size

The genome size of *D. turbinatus* and *H. hainanensis* was estimated with Illumina sequencing short reads through kmer method by using kmerfreq (version 5.0) (Marçais and Kingsford, 2011). From the kmer frequency distribution, the kmer depth was 28 and 35, and the total kmer number was 11 792 799 620 and 15 200 711 260, respectively. The genome size was estimated by the formula: genome size = K_num / k‐mer_depth.

### Genome assembly and annotation

NextDenovo (https://github.com/Nextomics/NextDenovo/) used Oxford Nanopore long reads to assemble the genome, including reads error correction with parameter ‘task=all (correct and assemble)’. We set read_cutoff = 1k, seed_cutfiles =10K while default parameters were used for other settings. At the genome polishing stage, NextPolish was used to correct the genome with three rounds of nanopore reads and thrice with Illumina sequencing reads.

There are three methods to evaluate the quality of the genome assembly. Firstly, the assembly N50 was more than 29Mb and 9Mb, respectively. The completeness of genome assembly was evaluated by BUSCO (version 2) with ‘eukaryota_odb9’ database (Additional file 2: Table S4) (Waterhouse *et al*., 2018). The accuracy used genome mapping rate to Illumina short reads by STAR (version 2.40) (Dobin *et al*., 2013).

Repeat elements were annotated using a combined strategy. We used both de novo and homolog‐based methods to find DNA transposon elements, retrotransposon elements and tandem repeats. For *ab initio* prediction, we used Piler‐DF, RepeatScout, MITE‐hunter, LTR_FINDER and RepeatModeler (version 1.0.8; http://www.repeatmasker.org/RepeatModeler/). Among them, Piler detected repeat elements such as satellites and transposons, RepeatScout identified all repeat classes, MITE‐hunter discovered miniature inverted‐repeat transposable elements (MITEs) from the genomic sequence, while LTR‐FINDER predicted the location and structure of full‐length LTR retrotransposons. All results from ab initio prediction were merged as a homolog database to identify repetitive sequences by RepeatMasker (http://www.repeatmasker.org). We used LAI (LTR Assembly Index) to evaluate the assembly continuity by evaluating the assembly of repeat sequences. LTR‐RT candidates were obtained using LTRharvest with parameters ‘‐minlenltr 100 ‐maxlenltr 7000 ‐mintsd 4 ‐maxtsd 6 ‐motif TGCA ‐motifmis 1 ‐similar 85 ‐vic 10 ‐seed 20 ‐seqids yes’ and LTR_Finder (version 1.0.7) with ‘‐D 20000 ‐d 1000 ‐L 700 ‐l 100 ‐p 20 ‐C ‐M 0.9’ (Ellinghaus *et al*., 2008). LTR_retriever was used to filter, unique and then obtain high‐confidence LTR retrotransposons with default parameters. Then, the genome LAI score was carried out by LAU program in the LTR_retriever with default parameter.

Gene models come from homology‐based prediction, de novo prediction and RNA‐seq‐based prediction. We used automated BRAKER2 to obtain accurate gene models which combined de novo and homology‐based predictions with GeneMark‐ES/ET and AUGUSTUS (Brůna *et al*., 2021). For training GeneMark‐TP and AUGUSTUS, we selected all Malvales proteins from the NR database (non‐redundant protein database). All protein‐coding genes were against several databases, including NR (plant database), SwissProt, KEGG (plant database), COG, InterProScan (using data from Pfam, PRINTS, SMART, ProDom and PROSITE) and GO by blastp (E‐value < 1e‐5).

### Pseudochromosome assembly based on Hi‐C data

Pseudochromosome validation involved three steps. First, HIC‐Pro was used to process the Hi‐C data from paired‐end raw reads to normalized contact maps with a resolution of 100 kb (Servant *et al*., 2015). The raw data with low quality, unmapped and invalid mapped paired reads were filtered out, and then, the assembly genome was integrated into a pseudochromosome‐scale assembly using the 3D de novo assembly (3D DNA) pipeline (Dudchenko *et al*., 2017). Juicebox Assembly Tools were used to view ‘.hic’ files from 3D DNA and further improve assembly by hand according to the contact maps (Durand *et al*., 2016).

### Gene family, phylogenomic analysis and estimation of divergence times

The genomes of species that were used for comparative genomics analysis were downloaded from public databases. OrthoFinder (v.1.1.8) was used to infer a homolog matrix of orthogroups (gene families) among these selected organisms (Emms and Kelly, 2019). Single copy gene families were used to construct phylogenetic trees based on maximum likelihood. In brief, multiple sequence alignment by MAFFT (v.7.310) for each single‐copy gene orthogroup, followed by gap position removal (only positions where 50% or more of the sequences have a gap are treated as a gap position). A maximum likelihood phylogenetic tree was constructed for each single‐copy gene family. The ASTRAL program was used to combine all single‐copy gene trees to a species tree with the multispecies coalescent model (Zhang *et al*., 2018). The Count software was implemented (with wagner parsimony algorithm) to analyse the orthogroups changes (such as gains, loss, expansions and contractions) of each lineage at every evolutionary node of the phylogenetic tree (Csűös, 2010). Divergence times between species were calculated using the MCMC tree program (http://abacus.gene.ucl.ac.uk/software/paml.html) implemented in Phylogenetic Analysis by Maximum Likelihood (PAML) (Yang and evolution, 2007). Expansion and contraction of the orthologous gene families were determined using CAFÉ software (De Bie *et al*., 2006).

### Analysis of genome synteny and whole‐genome duplication

We use the MCscan pipeline (https://github.com/tanghaibao/jcvi/wiki/MCscan‐(Python‐version)) and Circos (Krzywinski *et al*., 2009) for genome synteny. Ancient whole‐genome duplications were generated by command‐line tool WGD (Zwaenepoel and Van de Peer, 2019). Then, common evidence for ancient WGDs and synonymous substitutions per synonymous site (Ks) distributions were computed including whole‐paranome and one‐vs‐one ortholog in *D. turbinatus* and *H. hainanensis* and other related genomes (*G. raimondii*, *A. sinensis*, *C. capsularis*, *T. cacao* and *A. thaliana*).

To provide additional evidence of shared WGD event between the *D. turbinatus* and *H. hainanensis*, we extracted paralogous pairs of *D. turbinatus* and *H. hainanensis* genes derived from their respective WGDs and constructed phylogenetic trees. Firstly, we performed gene family cluster by using proteomes of *A. sinensis*, *A. thaliana*, *C. capsularis*, *D. turbinatus*, *H. hainanensis* and *T. cacao* to obtain orthogroups. We identified 4,539 and 3,098 gene pairs from *D. turbinatus* and *H. hainanensis* from Ks peak (Ks=0.22 ~ 0.35), respectively. 4,539 gene pairs of *D. turbinatus* were distributed in 3,578 orthogroups, and 3,098 gene pairs of *H. hainanensis* were distributed in 2,550 orthogroups. Then, we identified a total of 1,631 orthogroups containing both gene pairs of *D. turbinatus* and *H. hainanensis*. Next, 125 out of 1,631 orthogroups were randomly selected for phylogenetic study by using the IQtree (version 1.6.12) with parameter of ‘‐bb 10000 ‐alrt 5000 ‐nt AUTO’. Next, we reconciled ancestral gene events (duplications, losses and transfers) using a phylogenomic function in NOTUNG (version 2.9) by comparing the gene trees with the species tree. These trees were visualized with NOTUNG (Chen and Durand, 2000; Stolzer *et al*., 2012). Finally, we also checked the synteny of gene‐derived scaffold region between *D. turbinatus* and *H. hainanensis* in every phylogenetic tree. In summary, 108, 4, 5 out of 125 trees supported type I, II and III topologies, respectively, and a good synteny of gene pair‐derived scaffold region could be observed between *D. turbinatus* and *H. hainanensis* in each tree, thereby providing strong evidence of shared WGD event between both the species (Additional file 1: Figure S5).

To investigate *D. turbinatus* and *H. hainanensis* genome evolution, we do further genome‐wide duplications identification and classification by DupGen_finder with default parameters. We identified different modes of gene‐duplicated gene pairs and divided them into five types duplications: whole‐genome duplicates (WGD), tandem duplicates (TD), proximal duplicates (less than 10 gene distance on the same chromosome: PD), transposed duplicates (transposed gene duplications: TRD) and dispersed duplicates (other duplicates than WGD, TD, PD and TRD: DSD). The target species was *A. thaliana*, and the final gene number came from unique genes.

### Estimation of the divergence time

Divergence times in the phylogeny tree between each species were calculated using the MCMC tree program with ‐sampfreq 5000 ‐burnin 5000000 parameter. The sequential PHYLIP format nucleotide sequences and rooted phylogeny tree is derived from Figure 2. The divergence time was searched from TIMETREE (http://www.timetree.org/), *G. raimondii*‐ *D*. *zibethinus* divergence time (60‐77 MYA), *G. raimondii*‐ *T. cacao* divergence time (62‐85 MYA).

### Detection of key candidate functional genes

Based on the following criteria, all candidate genes were screened: firstly, candidate gene sequences were identical to collect query gene sequences gathered from previous studies or public databases, by BLAST (< 1e‐5); and (2) The candidate genes feature should be similar with the online functional annotation or Swissprot functional annotation query genes.

Regarding the identification of transcription factors (TFs), we used the HMMER search method for transcription factors. The Pfam website (https://pfam.xfam.org/) was used to download HMMER domain structure models for each transcription factor when as per the role of TAPscan v.2 database for TFs (https://plantcode.online.uni‐marburg.de/tapscan/). Preliminary TF candidate genes were collected for each species (< 1e‐5) by searching the HMM profile. Then, parts of genes were filtered if they are not the homologs according to their functional annotation of SwissProt (< 1e‐5). In the end, we filtered genes that contained a wrong domain under the TAPscan v.2 transcription factor database domain rules.

To identify genes involved in the terpenoid backbone biosynthesis pathway (Figure 4a), we collected the genes from *A. thaliana* that were documented in this pathway. Using these genes as a query sequence in BlastP, we predicted TPS genes with queries from *Atha*, *Vvin*, *Ptri* and rice, and the two Pfam domains, PF01397 and PF03936, were used to search by using HMMER. For BAHD identification, *Atha* members were used as queries to predict *A. sinensis*, *C. capsularis*, *D. turbinatus*, *H. hainanensis*, *T*. *cacao and V. vinifera* BAHD genes using BLASTP (1e−5). The CYP450 genes were searched by both domains, PF00067 and BLAST, with the queries from rice and *Atha*.

Finally, all searched candidate genes were used for phylogenetic analysis to distinguish the orthologs of corresponding functionally characterized genes. For each gene family, mafft‐7.310 used to align and Gblocks used to trim ambiguously aligned positions (Castresana, 2000). All the phylogenetic trees of functional genes were constructed by the maximum likelihood method with RAxML‐8.2.4 (Stamatakis, 2014).

### Transcriptome analysis of different tissues

The raw paired‐end RNA‐seq reads were filtered into clean data by FASTP (Chen *et al*., 2018). RSEM (https://deweylab.github.io/RSEM/) packages were used to estimate gene expression levels from clean reads. The transcriptome reads were mapped to the assembled genome by bowtie2 with default settings. We continue to identify the differentially expressed genes by DEseq2 (Love and Huber, 2014).

### Tissue‐specific Co‐expression Modules

To explore the dynamic changes of the genes and programs expressed, we performed weighted correlation network analysis (WGCNA) of gene expression in the leaf and stem (FPKM>1) (Langfelder and Horvath, 2008). When module displayed gene highly expressed in all 3 stem tissues and low expressed in all 3 leaf tissues, gene from this kind of module was selected for further co‐expression networks.

### Detection of metabolites by LC‐MS

The stem and leaf tissues were collected and stored in liquid nitrogen, then transferred to a freezer at −80°C. For the terpenoids detection, stem and leaf samples were preliminarily disposed of by using 2‐chlorophenylalanine (4 ppm) methanol. Next, samples with glass beads were put into the tissue grinder to grind for 90 s at 55 Hz. Following centrifugation at 12000 rpm at 4 °C for 10 min, take the supernatant, filter it through 0.22 μm membrane and transfer the filtrate into the detection bottle before LC‐MS analysis. Then, the sample extracts were analysed using an Ultra Performance Liquid Chromatography (UPLC) Vanquish (Thermo) and Q Exactive HF‐X system (Thermo). For the quantitative detection of phytohormones, stem and leaf tissue samples were used. The self‐construction database which is constructed by reference standards was used to perform qualitative analysis. Additionally, different concentrations of the standards were used to perform quantitative analysis.

## Conflicts of interest

The authors declare that they have no competing interests.

## Authors' contributions

H.L. and S.K.S. conceived, designed and supervised the project. J.W., L.C., J.Y., C.H., J.L. and Y.Z. provided resources and materials. S.W., H. Liang., S.K.S. and H.W. analysed the data. S.K.S., S.W. and H. Liang. wrote the paper. H.L., Y.L., T.M., M.L., L.L., Y.X. and M.L. revised the manuscript. All the authors read, revised and approved the final version of the manuscript.

## Supporting information


**Figure S1** Estimation of genome complexity of two Dipterocarpaceae trees.

**Figure S2** Hi‐C contact matrix visualization for chromosomes of two Dipterocarpaceae reference genome assemblies.

**Figure S3** Comparison of insertion dates of LTR‐RTs among *D. turbinatus*, *H. hainanensis* and *A. thaliana*.

**Figure S4** The phylogenetic analyses of Malvales based on nuclear and chloroplast genes.

**Figure S5** Phylogenetic evidence of shared WGD event between the *D. turbinatus* and *H. hainanensis*.

**Figure S6** Syntenic blocks between genomes.

**Figure S7** Phylogenetic tree of the TPS proteins.

**Figure S8** Chromosomal distribution of the important fragrance related genes in *D. turbinatus* and *H. hainanensis* genomes, respectively.

**Figure S9** Phylogenetic tree of the SABATH family, COMT and ICMT in the selected plants.

**Table S1** Statistics of contig and chromosome level genome assemblies of *D. turbinatus* and *H. hainanensis*.
**Table S2** Benchmarking Universal Single‐Copy Orthologs (BUSCO) evaluation of *D. turbinatus* and *H. hainanensis* genomes.
**Table S3** Annotation of repetitive sequences in *D. turbinatus* and *H. hainanensis* genomes.
**Table S4** Functional annotation of the *D. turbinatus* and *H. hainanensis* protein‐coding genes.
**Table S5** KEGG enrichment of expanded gene families of selected evolutionary nodes.
**Table S6** KEGG enrichment of unique gene families in Dipterocarpaceae.
**Table S7** Statistics of expanded genes number induced by different types of gene duplication way.
**Table S8** Aroma components in Dipterocarpaceae.
**Table S9** Genes involved in the 2‐C‐methyl‐D‐erythritol 4‐phosphate (MVA) and mevalonate (MEP) metabolism in selected plants.
**Table S10** Comparison of TPS gene number in selected plants.
**Table S11** Comparison of BAHD gene number in selected plants.
**Table S12** The candidate genes involved in the benzenoid/phenylpropanoid synthesis pathways in *D. turbinatus* and *H. hainanensis*.
**Table S13** The expression level of candidate genes involved in the terpenoid volatile organic compounds (VOCs) synthesis in wood and leaf tissue of D.
**Table S14** Comparison of gene number of SABATH family and SAM‐binding methyltransferase superfamily in selected plants.
**Table S15** Comparsion of transcription factors of selected plants.
**Table S16** Comparison of various phytohormones content between wood and leaf.
**Table S17** Genes involved in the fatty acid metabolism.
**Table S18** Protein ENHANCED PSEUDOMONAS SUSCEPTIBILITY in selected plants.
**Table S19** Statistics of resistance (R) genes.

## Data Availability

The data sets generated and analysed during the current study are available in the CNGB Nucleotide Sequence Archive (CNSA: https://db.cngb.org/cnsa ) under accession number CNP0002018.
