# Supplementary figures and images for: The chromosome‐scale genomes of Dipterocarpus turbinatus and Hopea hainanensis (Dipterocarpaceae) provide insights into fragrant oleoresin biosynthesis and hardwood formation
Source: Plant Biotechnol J. 2021 Dec 15;20(3):538–53. doi: 10.1111/pbi.13735 (PMC8882806; doi:10.1111/pbi.13735)

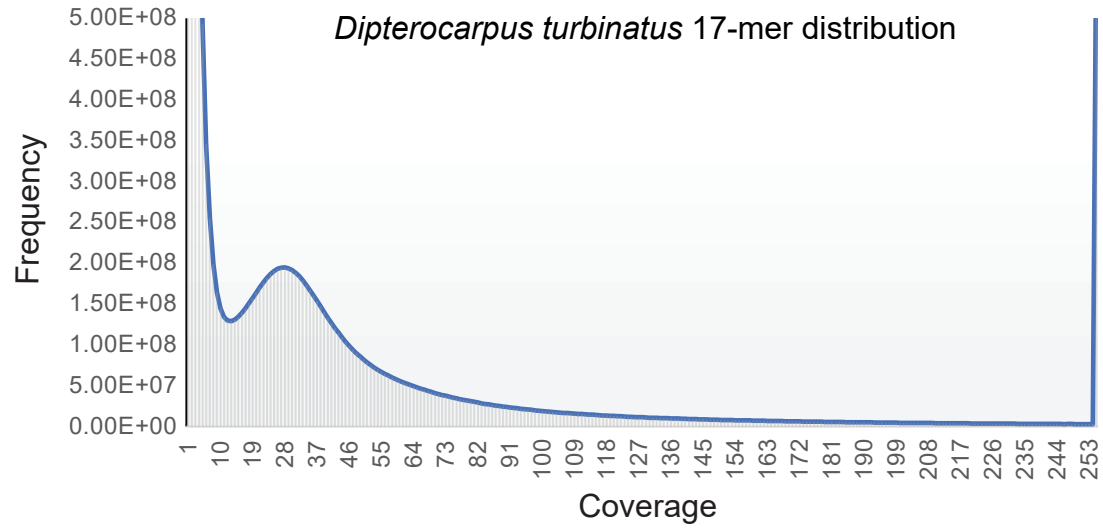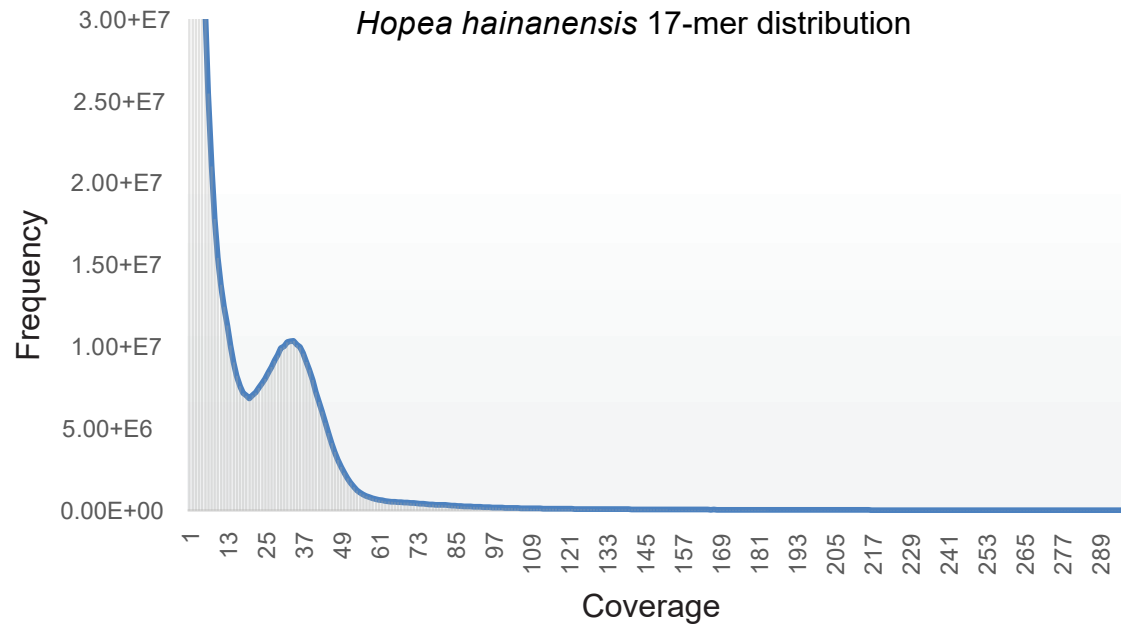

Supplement: Supplementary file 1 — Figure S1 Estimation of genome complexity of two Dipterocarpaceae trees. [file PBI-20-538-s009.pdf]

*D. turbinatus*

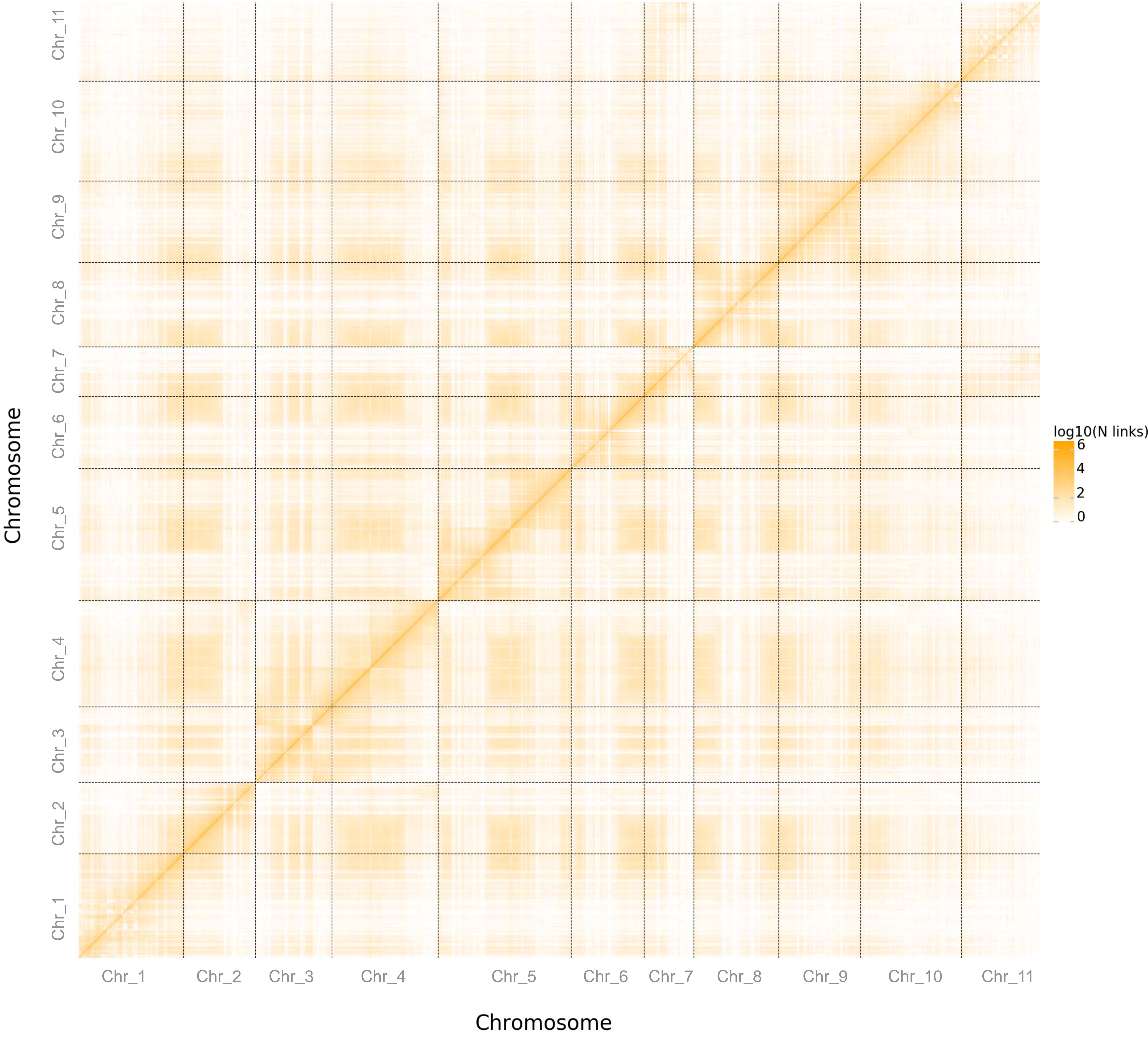

*H hainanensis*

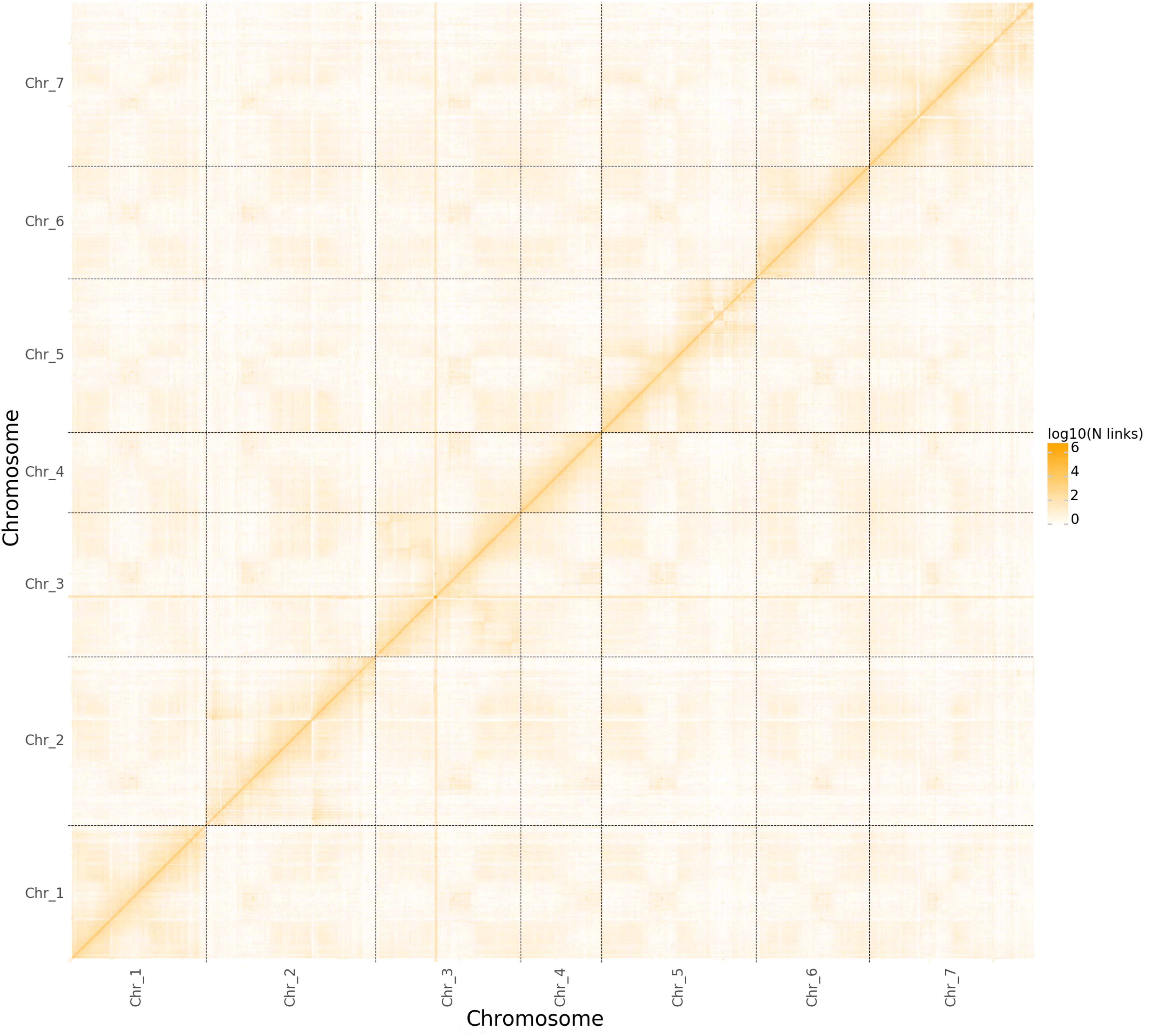

Supplement: Supplementary file 2 — Figure S2 Hi‐C contact matrix visualization for chromosomes of two Dipterocarpaceae reference genome assemblies. [file PBI-20-538-s007.pdf]

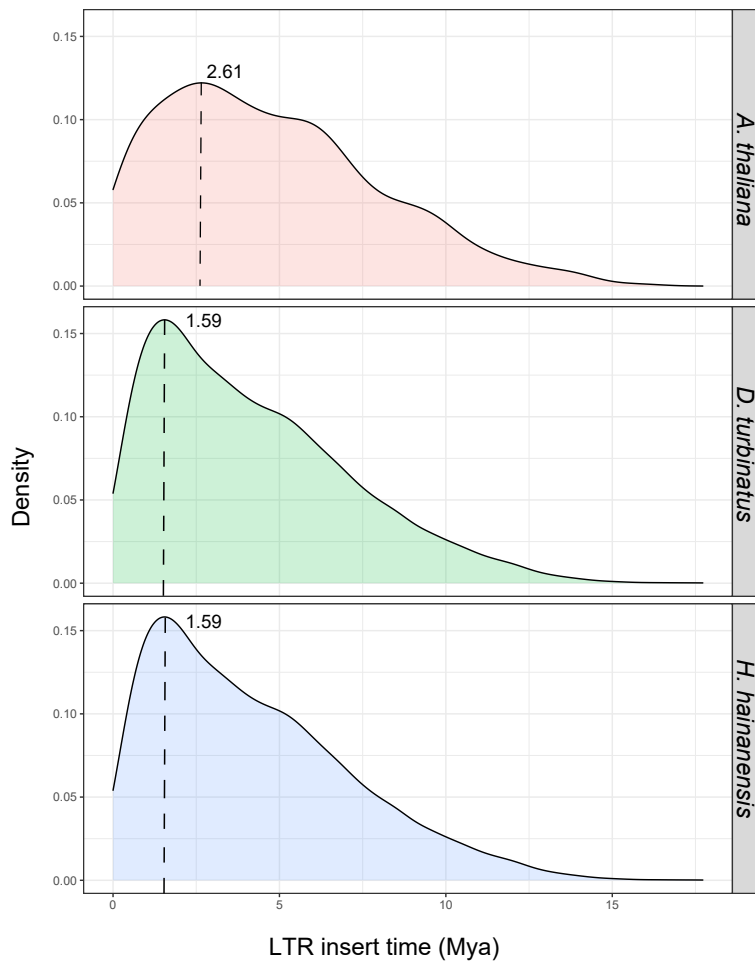

Supplement: Supplementary file 3 — Figure S3 Comparison of insertion dates of LTR‐RTs among D. turbinatus, H. hainanensis and A. thaliana. [file PBI-20-538-s008.pdf]

a.

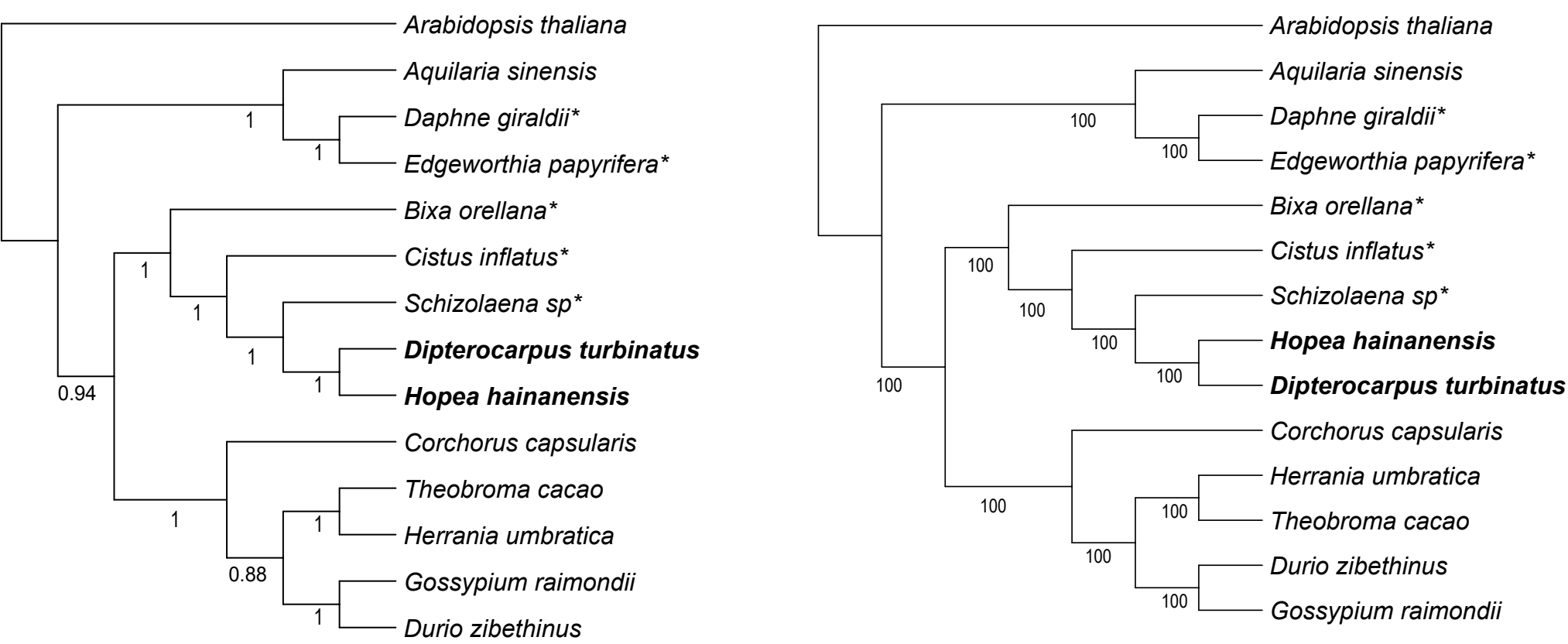

b.

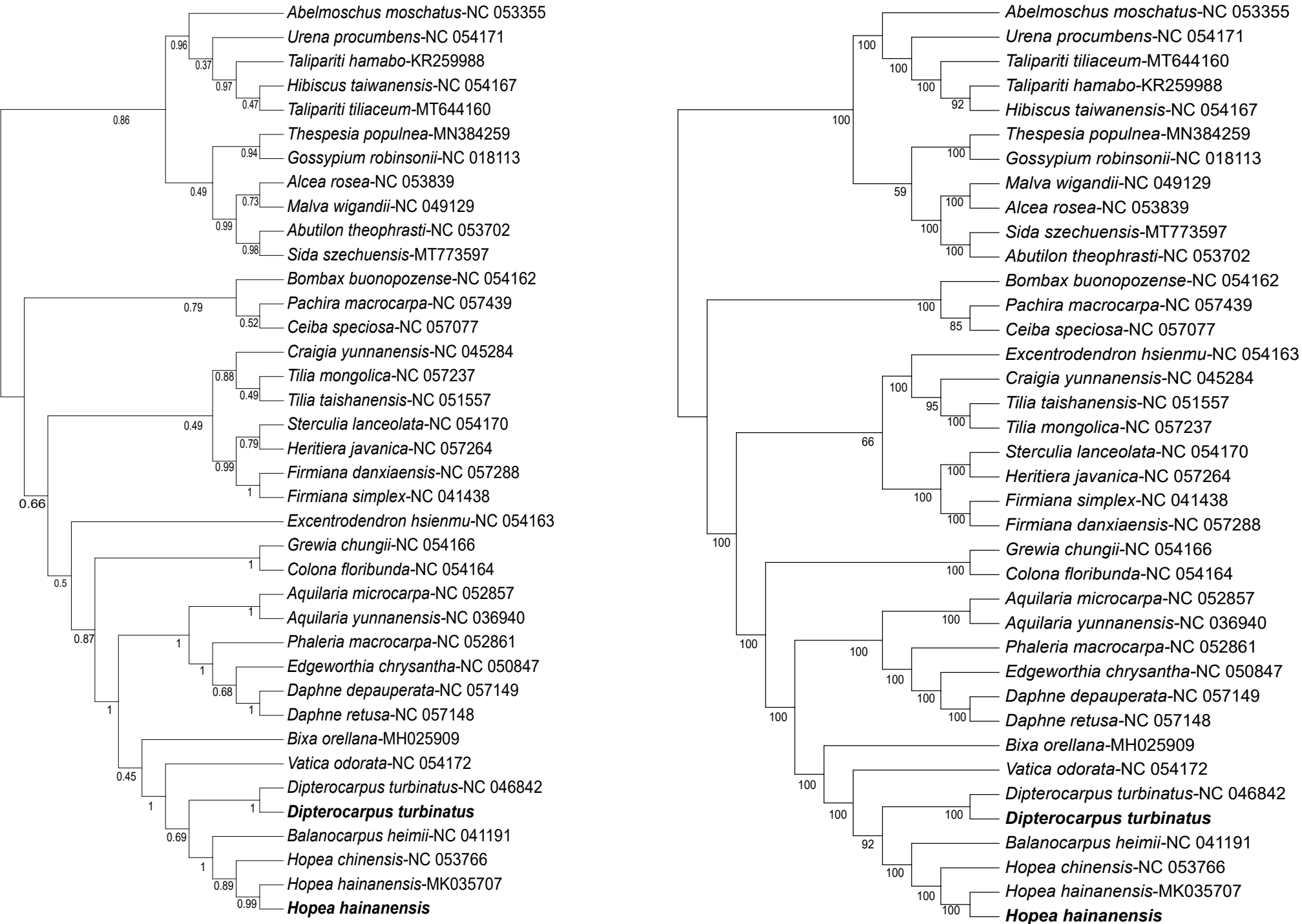

Supplement: Supplementary file 4 — Figure S4 The phylogenetic analyses of Malvales based on nuclear and chloroplast genes. [file PBI-20-538-s003.pdf]

a.

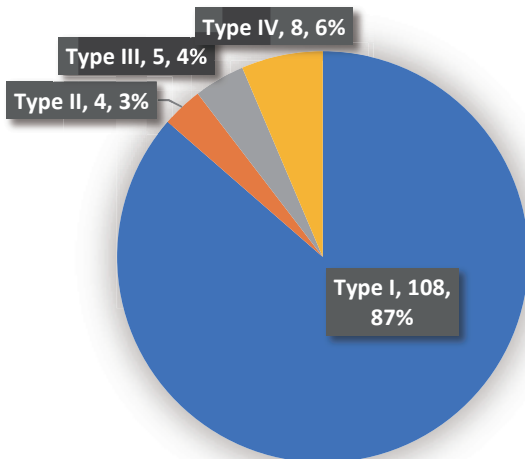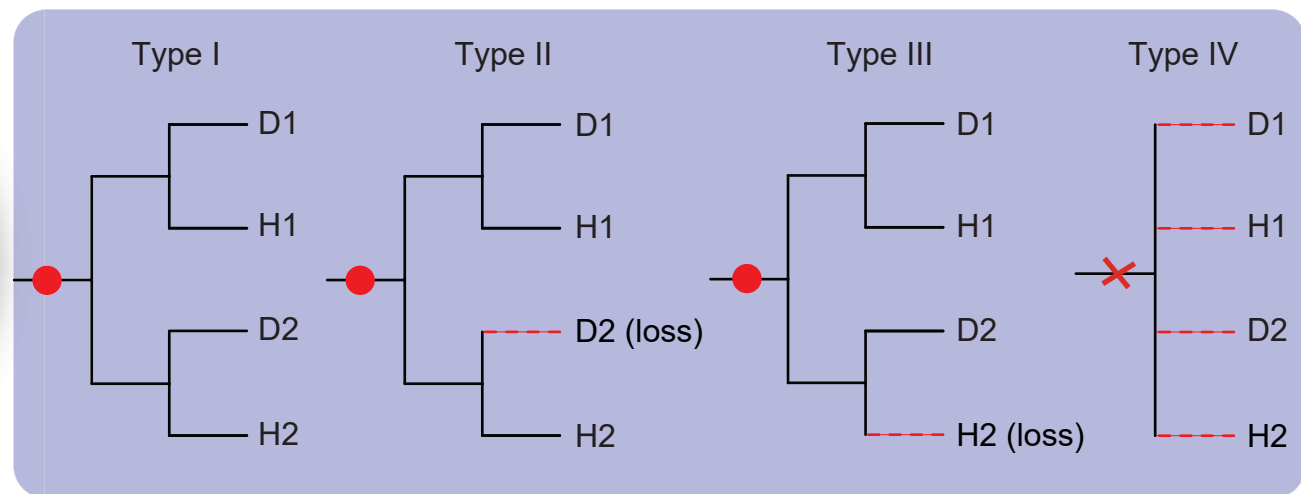

b.

Type I

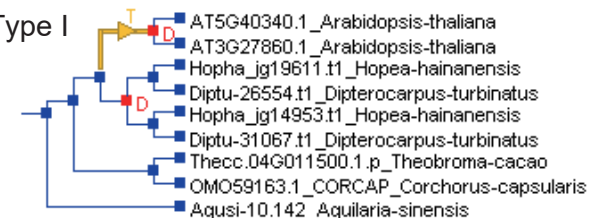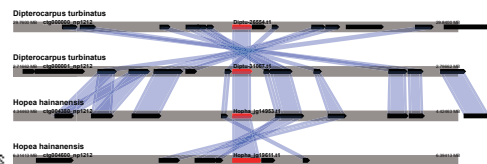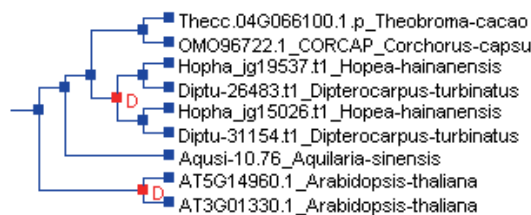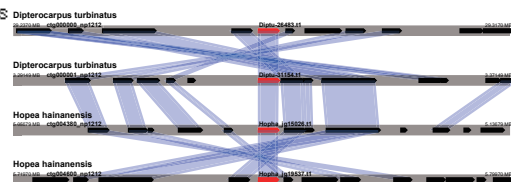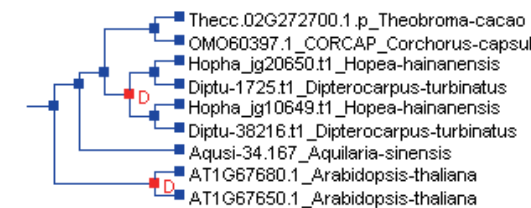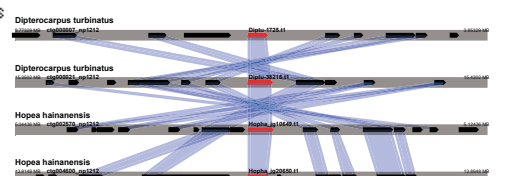

Type IV

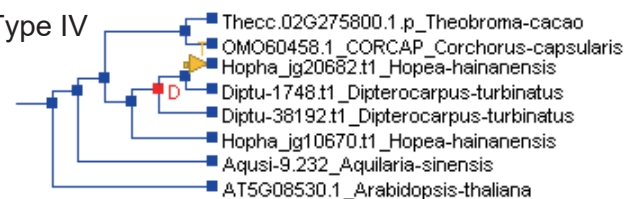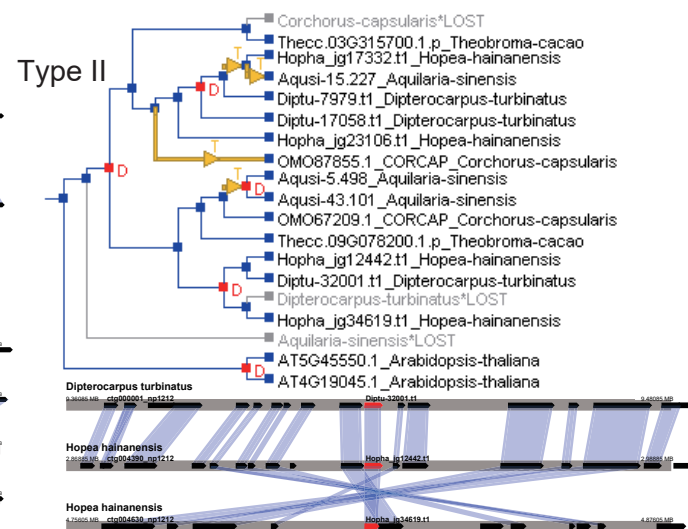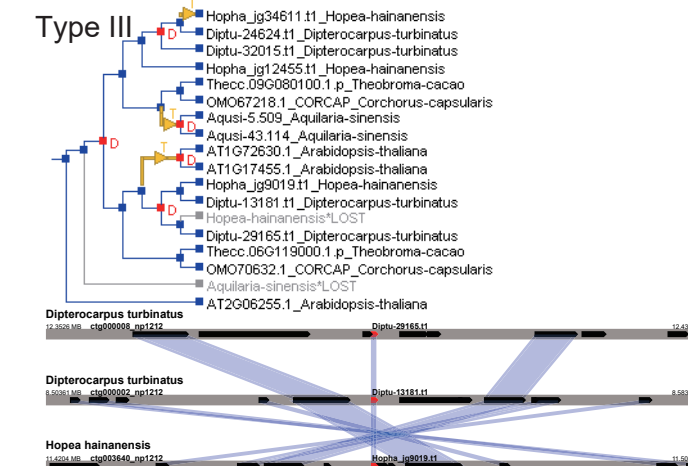

Supplement: Supplementary file 5 — Figure S5 Phylogenetic evidence of shared WGD event between the D. turbinatus and H. hainanensis. [file PBI-20-538-s002.pdf]

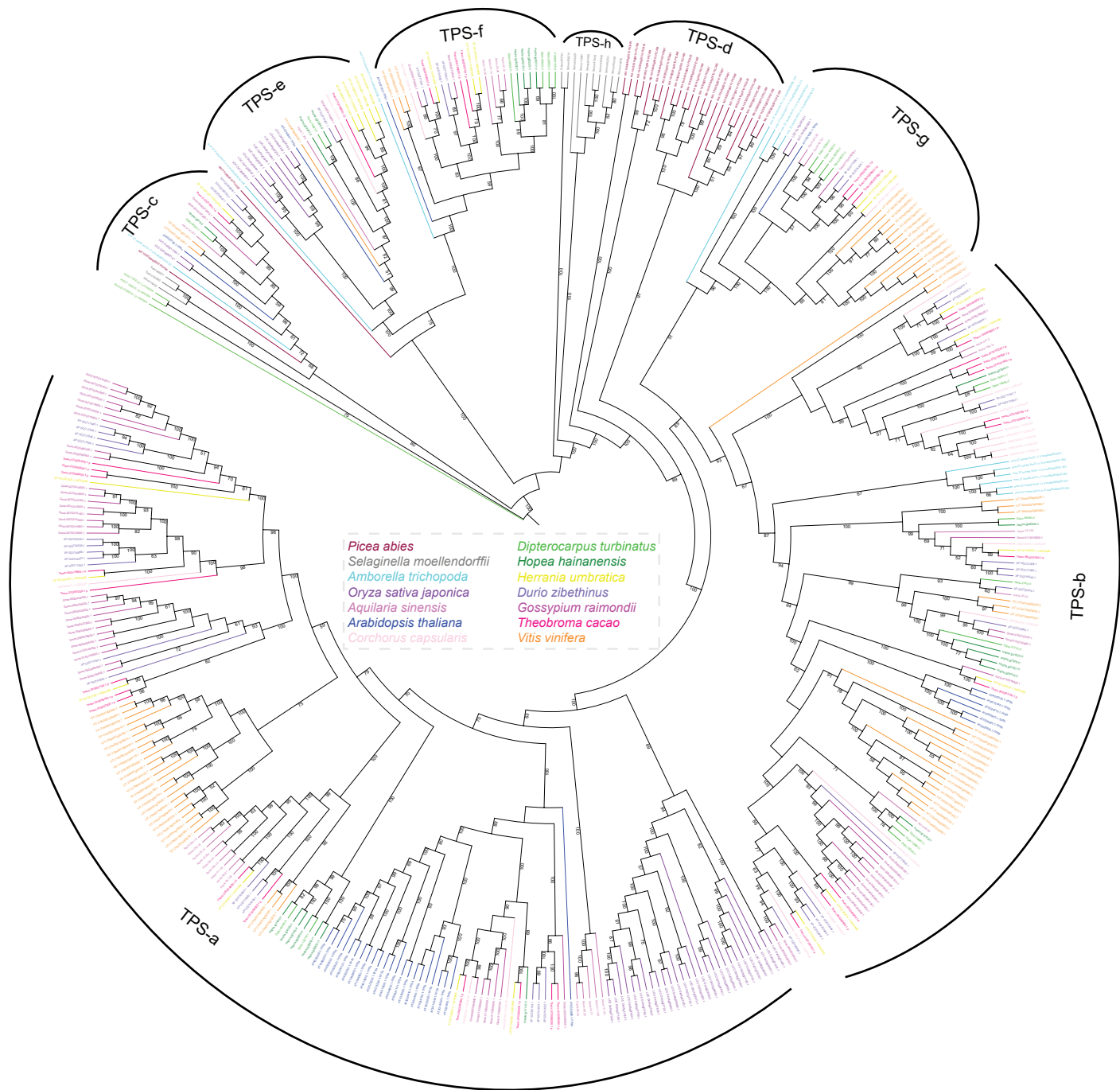

Supplement: Supplementary file 7 — Figure S7 Phylogenetic tree of the TPS proteins. [file PBI-20-538-s010.pdf]

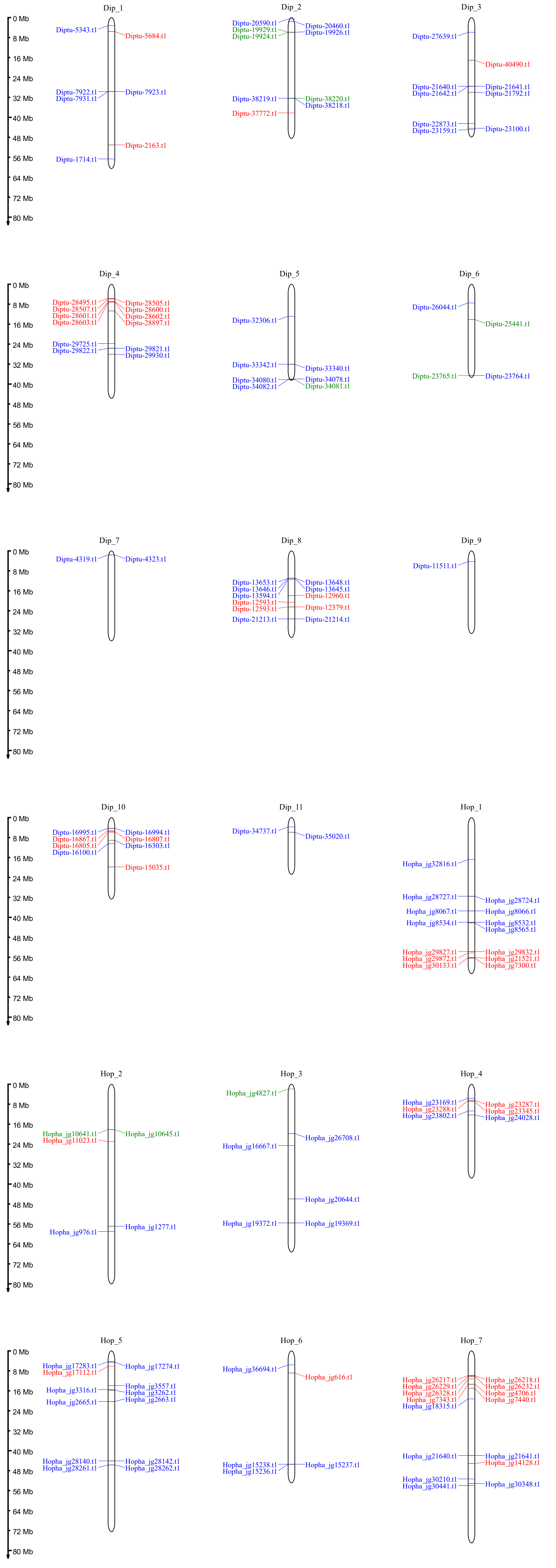

Supplement: Supplementary file 8 — Figure S8 Chromosomal distribution of the important fragrance related genes in D. turbinatus and H. hainanensis genomes, respectively. [file PBI-20-538-s001.pdf]

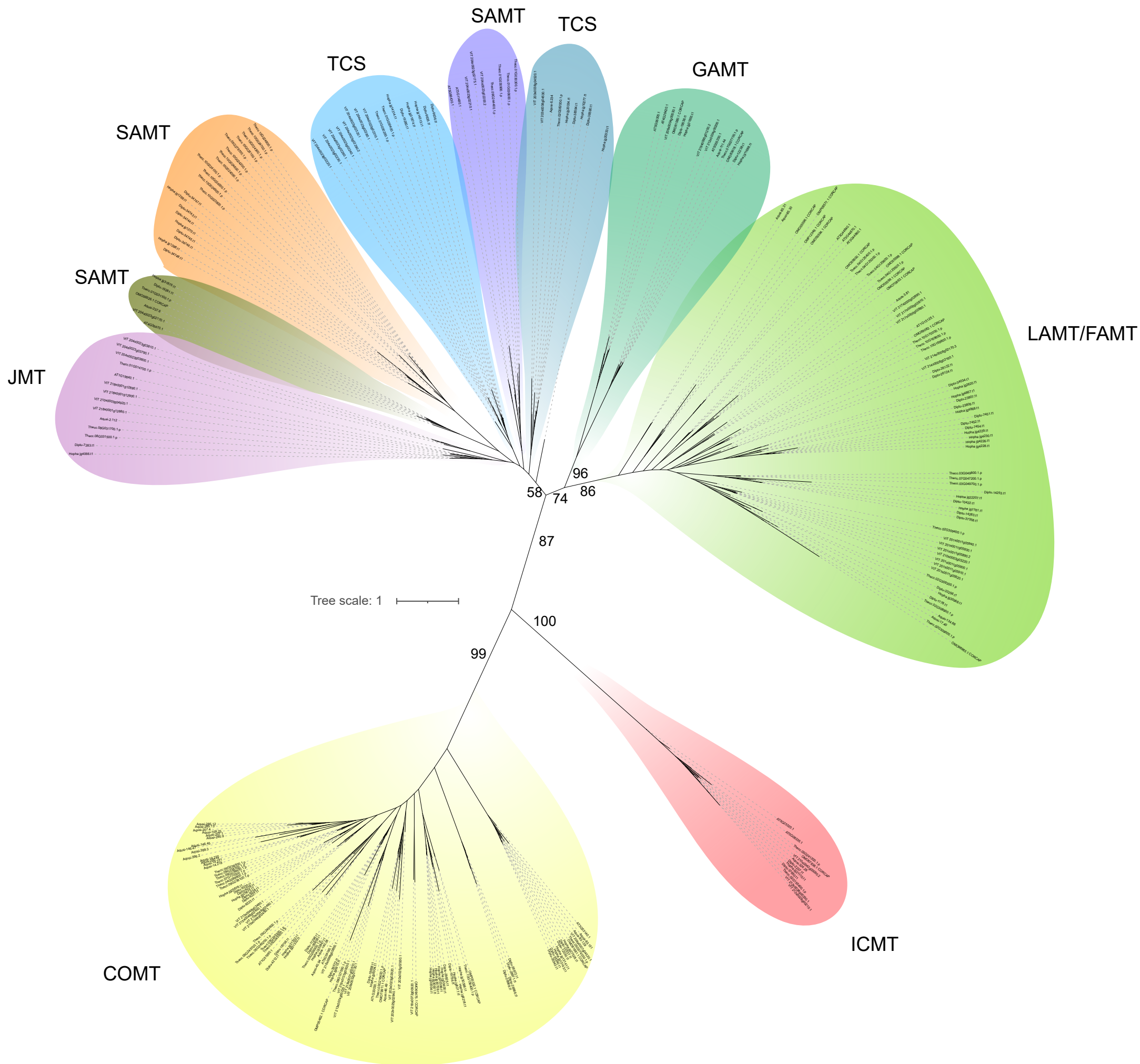

Supplement: Supplementary file 9 — Figure S9 Phylogenetic tree of the SABATH family, COMT and ICMT in the selected plants. [file PBI-20-538-s004.pdf]
