# Supplementary material for: The chromosome‐scale genomes of Dipterocarpus turbinatus and Hopea hainanensis (Dipterocarpaceae) provide insights into fragrant oleoresin biosynthesis and hardwood formation
Source: Plant Biotechnol J. 2021 Dec 15;20(3):538–53. doi: 10.1111/pbi.13735 (PMC8882806; doi:10.1111/pbi.13735)
Supplement: Supplementary file 6 — Figure S6 Syntenic blocks between genomes. [file PBI-20-538-s005.pdf]

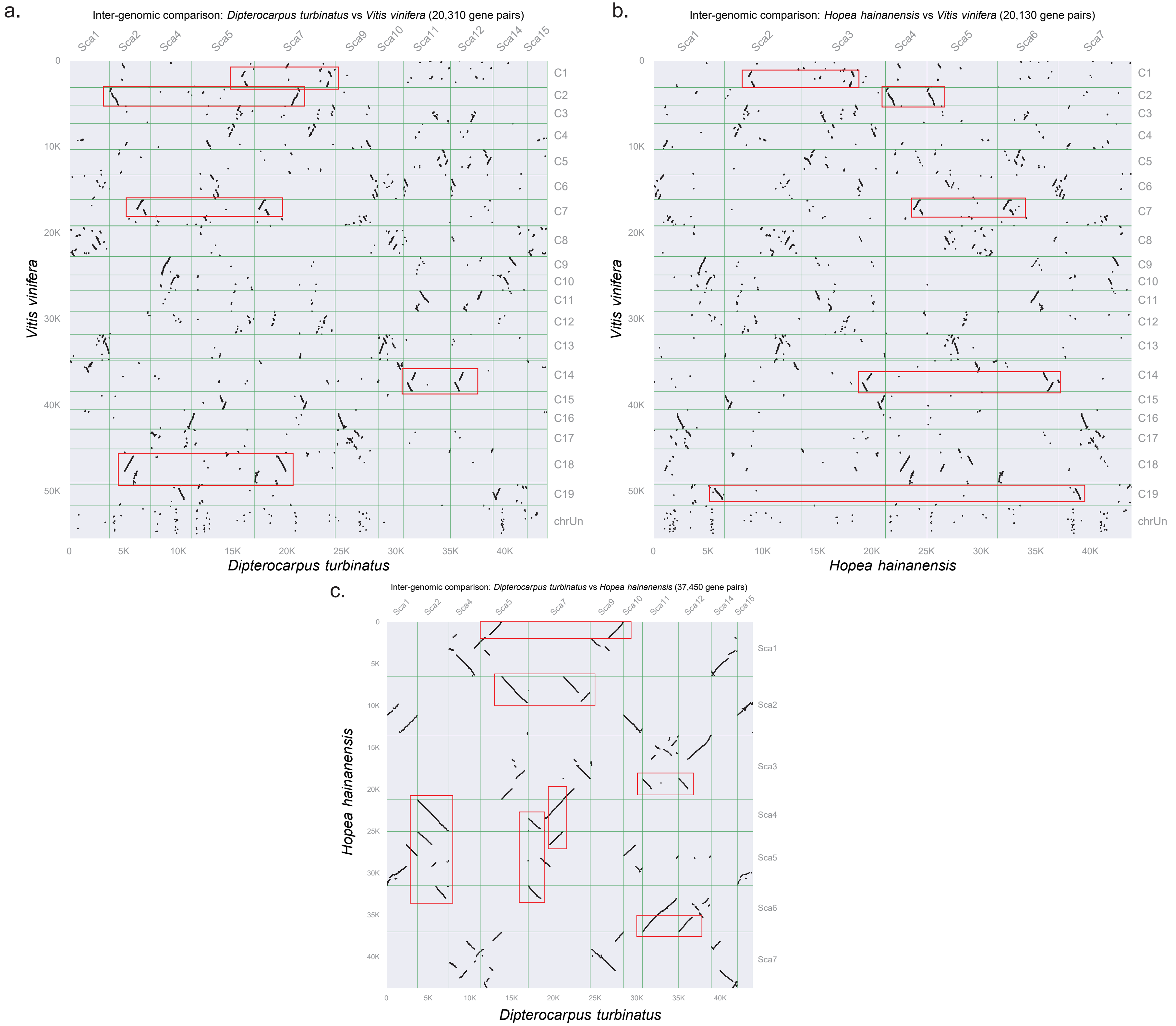

a.
Inter-genomic comparison: *Dipterocarpus turbinatus* vs *Vitis vinifera* (20,310 gene pairs)
Sca1 Sca2 Sca4 Sca5 Sca7 Sca9 Sca10 Sca11 Sca12 Sca14 Sca15
C1 C2 C3 C4 C5 C6 C7 C8 C9 C10 C11 C12 C13 C14 C15 C16 C17 C18 C19 chrUn
0 10K 20K 30K 40K 50K
0 5K 10K 15K 20K 25K 30K 35K 40K
Vitis vinifera
Dipterocarpus turbinatus
b.
Inter-genomic comparison: *Hopea hainanensis* vs *Vitis vinifera* (20,130 gene pairs)
Sca1 Sca2 Sca3 Sca4 Sca5 Sca6 Sca7
C1 C2 C3 C4 C5 C6 C7 C8 C9 C10 C11 C12 C13 C14 C15 C16 C17 C18 C19 chrUn
0 10K 20K 30K 40K 50K
0 5K 10K 15K 20K 25K 30K 35K 40K
Vitis vinifera
Hopea hainanensis
c.
Inter-genomic comparison: *Dipterocarpus turbinatus* vs *Hopea hainanensis* (37,450 gene pairs)
Sca1 Sca2 Sca4 Sca5 Sca7 Sca9 Sca10 Sca11 Sca12 Sca14 Sca15
Sca1 Sca2 Sca3 Sca4 Sca5 Sca6 Sca7
0 5K 10K 15K 20K 25K 30K 35K 40K
0 5K 10K 15K 20K 25K 30K 35K 40K
Hopea hainanensis
Dipterocarpus turbinatus
